# Health-seeking behaviour, referral patterns and associated factors among patients with autoimmune rheumatic diseases in Ghana: A cross-sectional mixed method study

**DOI:** 10.1371/journal.pone.0271892

**Published:** 2022-09-12

**Authors:** Maame-Boatemaa Amissah-Arthur, Anna Gyaban-Mensah, Vincent Boima, Ernest Yorke, Dzifa Dey, Vincent Ganu, Charles Mate-Kole

**Affiliations:** 1 Department of Medicine and Therapeutics, University of Ghana Medical School, College of Health Sciences, University of Ghana, Accra, Ghana; 2 Department of Psychiatry, Korle Bu Teaching Hospital, Accra, Ghana; 3 Department of Medicine and Therapeutics, Korle Bu Teaching Hospital, Accra, Ghana; 4 Center for Ageing Studies, College of Humanities, University of Ghana, Legon, Ghana; 5 Department of Psychology, College of Humanities, University of Ghana, Legon, Ghana; King’s College London, UNITED KINGDOM

## Abstract

**Background:**

Challenges exist in the diagnosis and management of autoimmune rheumatic diseases in low and middle income countries due to factors, such as poverty and under-resourced healthcare infrastructure. Furthermore, other contributory factors such as societal, cultural and religious practices influence health seeking behaviour which has a bearing on access and delivery of healthcare.

**Objectives:**

To examine the health seeking behaviour and referral patterns of Ghanaian patients with autoimmune rheumatic diseases and assess the associated factors that influence these.

**Method:**

A cross-sectional study using an explanatory sequential mixed method design was carried out in a Rheumatology clinic at a national referral centre. 110 participants were purposively recruited for the quantitative phase. The qualitative phase comprised 10 participants for in-depth interviews and 10 participants for a focus group discussion. Analysis using descriptive statistics, t-tests and logistic regression models were performed. Transcripts generated from the interviews and focused group discussion were analysed using thematic analysis.

**Results:**

Median duration from onset of symptoms until seeking help was 1 week (IQR = 12); from seeking help until obtaining a final diagnosis was 12 months (IQR = 33). Multiple factors determined the choice of first facility visited, X^2^ (12, N = 107) = 32.29, p = .001. Only twenty-one participants (19.6%) had knowledge of their disease prior to diagnosis. Education predicted prior knowledge [OR = 2.6 (95% CI = .66–10.12), p < .021]. Unemployed participants had increased odds of seeking help after a month compared to those who were employed [Odds ratio = 2.60 (95% CI = 1.14–5.90), p = .02]. Knowledge of autoimmune rheumatic diseases was low with multiple causative factors such as biomedical, environmental and spiritual causes determining where patients accessed care. Forty (36.4%) participants utilised complementary and alternative treatment options.

**Conclusion:**

We observed that knowledge about autoimmune rheumatic diseases among Ghanaian patients was low. Patients sought help from numerous medical facilities, traditional healers and prayer camps often contributing to a delay in diagnosis for most patients. This was influenced by individual perceptions, cultural beliefs and socioeconomic status. Active awareness and educational programmes for the public and healthcare workers are required, as well as strategic planning to integrate the biomedical and traditional care services to enable earlier presentation, accurate diagnosis and better clinical outcomes for the patients.

## Introduction

Autoimmune rheumatic diseases (ARDs) are a group of disorders characterized by loss of immune tolerance, autoantibody production, organ inflammation and damage. If they are not diagnosed and managed early they can result in high morbidity and mortality. Some examples include rheumatoid arthritis (RA), systemic lupus erythematosus (SLE), systemic sclerosis (SSc), inflammatory myositis, mixed connective tissue disease (MCTD) and systemic vasculitides.

Awareness and knowledge of these conditions in low and middle income countries have been steadily increasing in recent times [1, 2], however they are still faced with challenges in disease management and high case fatality rates due to factors such as poverty [3], under-resourced healthcare infrastructure and limited clinical expertise [4]. For example, SLE data from high income countries show significant improvement in survival rates over the past decades [5], however amongst people of African, Caribbean and Hispanic descent in western countries [6], and native Africans in sub Saharan Africa mortality rates remain high [7, 8]. Additionally patient-dependent factors, such as perception of illness and external influence determine their health seeking practices and are contributory factors [9, 10].

Health beliefs which are determined by societal, religious and cultural practices, influence illness behaviour [11]. Specifically, in some African countries, there are strong “spiritual and supernatural” beliefs that explain the unknown and the basis of illness. As a result, orthodox medical interventions are not usually considered the first option in seeking help leading to a delay in access and delivery of appropriate health care [12, 13]. These spiritual and cultural beliefs are pervasive in Ghana, however there is lack of knowledge on patient-related factors that are associated with the health-seeking behaviour among patients with autoimmune rheumatic diseases in Ghana.

The purpose of the present study is to explore the health seeking behaviour and referral patterns of Ghanaian patients with autoimmune rheumatic diseases and assess the associated factors that influence these.

## Methods

### Study design and setting

A cross-sectional study with an explanatory sequential mixed methods approach was used in this study. Quantitative data was collected and analysed at the initial phase followed by a second phase of qualitative data collection to further explain findings obtained from the initial phase of the study [14]. Participants with ARDs were recruited from the outpatient Rheumatology Clinic of Korle Bu Teaching Hospital, a tertiary referral centre located in the capital city Accra, from November 2018 to July 2019.

### Study population and sampling procedure

Patients who were 18 years and older and had a confirmed diagnosis of an autoimmune rheumatic disease using standardised diagnostic or classification criteria specific for the condition were included in the study [ACR/EULAR RA Classification criteria 2010; ACR SLE Classification criteria 1997; Bohan and Peter Criteria 1975; EULAR/ACR Systemic Sclerosis Classification Criteria 2013; Mixed Connective Tissue Disease according to Alarcón-Segovia Criteria 1996]. Patients who could not complete the questionnaire or interview due to a language barrier, nor provide written informed consent were excluded from the study.

A multistage sampling technique was used to select the participants. Firstly, purposive sampling technique was used to select old and new referral cases who met the inclusion criteria for the study.

At the time of conducting the study, no prevalence data for autoimmune rheumatic diseases were available for the Ghanaian population. Additionally, there is paucity of data generally within Africa for the individual disorders. The sample size for this study was calculated using prevalence data from other African studies. A meta-analysis of general rheumatic diseases across numerous general and rheumatology units showed the pooled prevalence of SLE to be 1.7% [15]. Meta-analysis from a South African urban population showed prevalence of 2.5% [1]. There was a lower incidence of reporting of the other ARDs. Worldwide, RA is reported to be the most common autoimmune inflammatory rheumatic disease. The sample size for the study was thus calculated using the higher RA prevalence of 2.5%. The sample size was computed using Cochran’s formula and then a modification for small populations was done [16].

*n*≥[(*Z*_(1−α/2)_)^2^*p*(1−*p*)]/E^2^

Where n = recommended sample size

*Z*_(1−α/2)_ = Z value for confidence level chosen (95%) = 1.96

P = prevalence of rheumatic conditions from previous study = 2.5%

E = margin of error = 5%

n = n_0_/(1+ (n_0−_1)/N)

n_0_ = Initial recommended sample size

n = modified sample size

N = Population size

The initial sample size calculation was 38. The total number of all cases of ARDs at Korle Bu Teaching Hospital at the start of the study was 763. Therefore, the adjusted minimum sample size required for the study was 37. The final number of patients recruited for the study was 110, which was sufficient, taking into account an anticipated non-response rate of 20%.

Following the quantitative phase of the study, 20 participants were consecutively sampled from the initial pool of 110 participants. Phone calls were made and based on their availability and willingness to participate for the second phase of the study, they were invited to participate on a fixed day and time. The in-depth interviews were conducted first on 10 participants. This was followed by one focus group discussion to complement the individual interviews; also comprising 10 participants (phenomenological research sample size range from 3 to 4 individuals to 10 to 15) [14]. A flowchart summarizing participant recruitment into the quantitative and qualitative arms of the study is shown in S1 Fig.

### Data collection

Quantitative data was collected using a semi-structured questionnaire administered by a trained research assistant. The questionnaire was presented in English. If the patient was unable to read or communicate in English, the interviewer or a translator proceeded in a chosen dialect. The questionnaire was pre-tested in ten patients from the rheumatology clinic to assess the language and content, timing and subsequently, adjustments were made to ensure that the questions were well understood. These patients were not included in the current study. Data of the final 110 participants were analysed. See Supporting information for the questionnaires (S1 Appendix).

Baseline socio-demographic characteristics including age, sex, religion, education, employment, and geographical location were recorded. Clinical details regarding type and duration of symptoms, diagnosis, facilities visited and diagnosing facility were recorded. The facilities were categorised into non-specialist (pharmacies, primary care clinics/hospitals); specialist rheumatology services; and non-medical facilities (churches, prayer camps and herbal medical centres).

The measure for “knowledge of disease” had 5 items. Four of the items had multiple coded responses and one had a response scale “Yes and No”. Items included: Have you ever (previously) heard of your illness/condition prior to diagnosis? What do you believe to be the basis of your illness? The measure for “perception of disease” consisted of 6 items. Two of the items had a “Yes or No” response scale while the rest had multiple coded responses. Some of the items included: What do you perceive others think of you and your illness?

Nafkam International Complementary and Alternative Medicine Questionnaire (I-CAM-Q) was used to assess contemporary and alternative medicine use among patients [17]. The questionnaire has 4 sections, namely the facilities of health care providers visited, complementary and/or alternative treatments received, use of herbal medicine and dietary supplements and use of various self-help practices.

Qualitative data was collected by clinical psychologists trained in qualitative methodology, as a second arm of the study to enable further exploration of questions not easily answered by the quantitative methods and record perceptions of the participants in the context of culture [18]. A semi-structured interview guide designed for a focus group discussion and in-depth interviews was used and each interview lasted between 40–50 minutes. The interview guide had open-ended questions with probes and comprised five domains: knowledge, belief, attitudes/practices, stigma and general impact of ARDs on patient. The interview guide was pre-tested using 4 dialysis-dependent, chronic kidney disease patients for validity. The language and content of the guide were reviewed after pilot testing for easy understanding and administration during data collection. The interviews were audio-recorded and transcribed for analysis. See Supporting information for the Interview guide (S2 Appendix).

### Data analysis

#### Quantitative analysis

Data collected were entered and cleaned using SPSS version 22.0. Data analyses were conducted using Stata version 15 (StataCorp, College Station, TX, USA). Descriptive statistics were used for frequency counts and percentages of participant characteristics. The p-values of Chi square and Fisher’s exact tests of association were recorded for the association between the socio-demographic variables, knowledge and perception. Student t-tests were used to assess differences in duration of experiencing symptoms as well as delay in diagnosis, across socio-demographic characteristics and diagnoses. Logistic regression models were used to determine significant predictors of knowledge and access to health care. Statistical significance was determined at 95% confidence level (S3 Appendix).

#### Qualitative interpretation

The qualitative findings were reported in accordance to the consolidated criteria for reporting qualitative studies (COREQ). See Supporting information for the COREQ checklist (S4 Appendix). Participants’ responses were transcribed verbatim and analysed using thematic analysis. This involved a) familiarisation of the data, b) generating initial codes, c) searching for themes, d) reviewing themes, e) defining and naming themes and f) producing the report [19]. Illustrative quotations were recorded and included. Two experts in qualitative research and analysis independently coded the transcripts and later compared themes that were generated. Discussions occurred where there were discordant themes generated and consensus was reached. Transcripts from the interviews are available in S5 Appendix.

### Ethics statement

This research was conducted in accordance with the Declaration of Helsinki on human subjects. Ethical approval was obtained from the Ethical and Protocol Review Committee of the College of Health Sciences, University of Ghana (CHS-Et/M.1-P2.10/2018-2019).

## Results

### Quantitative results

Majority of the participants were female (86.4%) and the mean age was 36.8 years (SD = 14.6 years). The most common ARD was RA (45.5%) followed by SLE (36.4%) and there was a statistically significant difference between the mean age of onset for RA patients and SLE patients, 36.8 years (SD = 15.0) and 25.1 years (SD = 9.4), p <0.001, respectively. The other socio-demographic characteristics are recorded in Table 1.

**Table 1:** Socio-demographic and clinical characteristics of study participants.

**Characteristics**	**Frequency (n = 110)**	**Percentage (%)**
**Age (yrs)**		
Ave (SD) = 36.8 (14.6)		
Below 29	45	40.9
30 to 45	37	33.6
Above 45	28	25.5
**Gender**		
Female	95	86.4
Male	15	13.6
**Marital Status**		
Married/ Co-Habiting	48	43.6
Single/Never Married	53	48.2
Divorced/Separated	4	3.6
Widow/Widower	5	4.5
**Religion**		
Christianity	99	90
Islam	11	10
**Educational Level**		
Ave (SD) (yrs) = 13.1(3.6); Range 0 to 18 years		
Junior secondary and below	21	19.1
Senior secondary/O’/A’ Level	36	32.7
Tertiary	53	48
**Occupation**	(n = 109)	
Employed	56	51.4
Unemployed	53	48.6
**Region**		
Greater Accra	85	77.3
Central	3	2.7
Ashanti	6	5.5
Northern	1	0.9
Volta	2	1.8
Eastern	9	8.2
Western	3	2.7
Brong Ahafo	1	0.9
Upper West	0	0
Upper East	0	0
**Diagnosis**		
RA	50	45.5
SLE	40	36.4
MCTD	16	14.5
SSc	4	3.6

#### Time to diagnosis

The median duration from onset of symptoms until diagnosis was 18 months (IQR = 43.94) across all disease groups. Once symptoms began, there was a variation in the length of time it took participants to seek any form of help such as the pharmacist, hospital care or non-medical care. The median time was 0.23 months (IQR = 3) [i.e. 1 week], with a minimum of less than one week and maximum duration of 204 months. The median duration from seeking help at the first facility visited to the final diagnosis was 12 months (IQR = 33). In the two largest disease groups, the mean duration of symptoms until diagnosis for RA patients was 36.0 months (SD = 54.3) and 17.5 months (SD = 28.8) for SLE patients, respectively.

Duration of symptoms were categorised as “*less than a month*” and “*one month and over*” based on the median. Employment status was significantly associated with the categories of duration of symptoms, *X*^2^ (4, *N* = 102) = 10.017 *p* = .040. Logistic regression analysis was conducted which showed an increased odds of *seeking help* after a month among those who were unemployed as compared to those who were employed [Odds ratio = 2.60 (95% CI = 1.14–5.90), p = .02].

#### Type of facility visited

**Fig 1:**
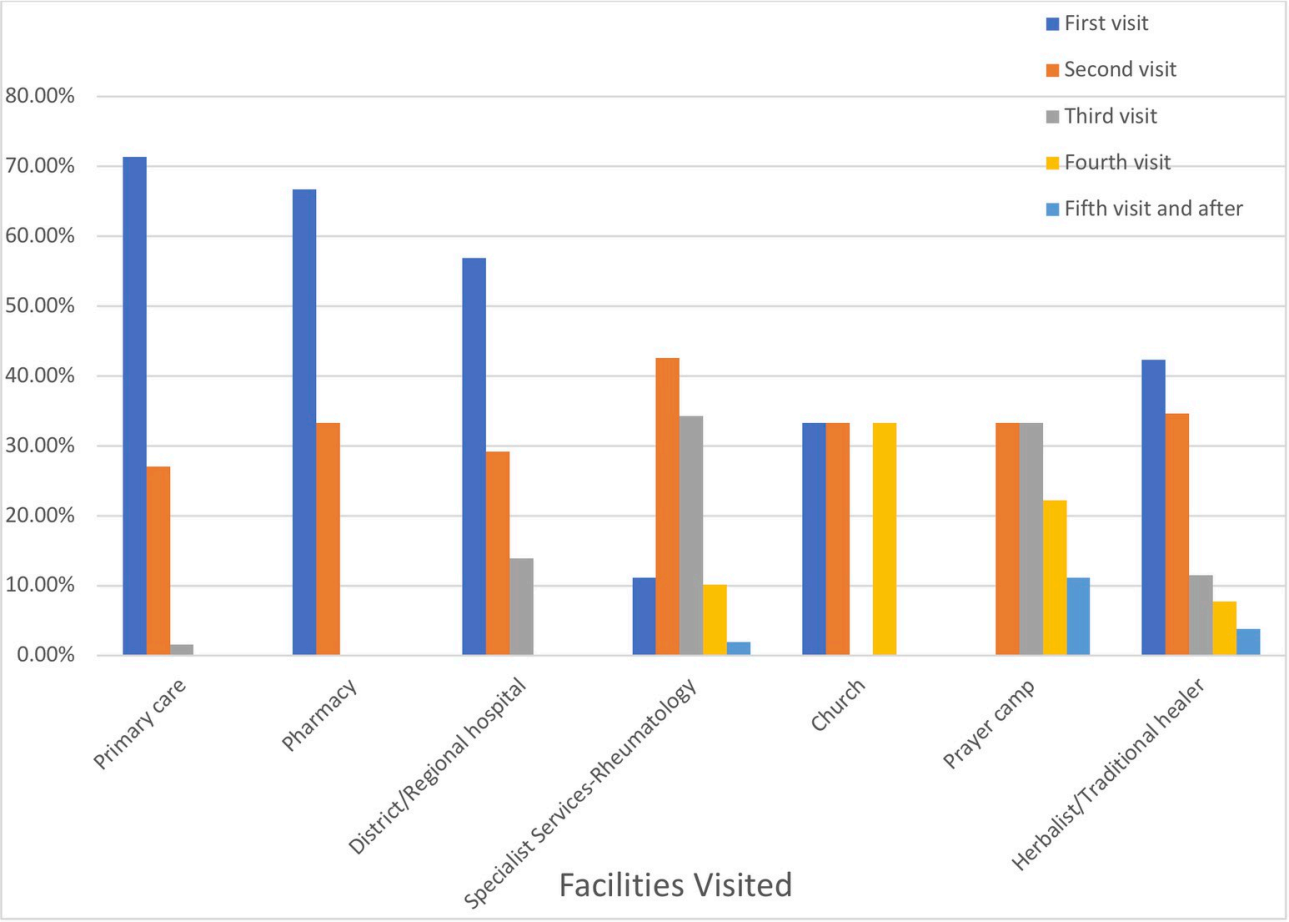
Distribution and order of facilities visited by attendance.

At their first point of contact, 90/110 (81%) participants visited non-specialist centres. These were primarily pharmacies to buy over-the-counter medication for self-administration; general practitioners and district hospitals for consultations. Fewer participants presented to the specialist rheumatology centre as the first point of care (9%), with the majority (77%) presenting there as a later option. 11 (10%) participants attended churches/prayer camps and herbal medical centres first. A further breakdown of the attendance pattern is shown in Fig 1.

Participants reported that their choice of seeking help was influenced by the type of symptoms they experienced [examples reported were over-the-counter analgesia for arthralgia; prayer camp for psychotic presentation of SLE]; proximity of the facility to their home or place of work; recommendation from a third party; and previous or current attendant of the facility. *X*^2^ (12, *N* = 107) = 32.29, *p* = .001. See Table 2.

**Table 2:** Analyses of association between socio-demographic characteristics of participants and first facility visited.

Characteristics N (%)	Total	Non-specialist	Specialist	Non-Medical	p-value*
**Age Category**	110				.593
29 years and below	45	38 (84.4)	4 (8.9)	3 (6.7)	
30 to 45 years	37	30 (81.1)	4 (10.8)	3 (8.1)	
Above 45 years	28	21 (75.0)	2 (7.1)	5 (17.9)	
**Gender**	110				.068
Male	15	10 (66.7)	1 (6.7)	4 (26.7)	
Female	95	79 (83.2)	9 (9.5)	7 (7.4)	
**Marital Status**	110				.747
Married/ co-Habiting	48	37 (77.1)	5 (10.4)	6 (12.5)	
Single/Never Married	53	45 (84.9)	4 (7.5)	4 (7.5)	
Divorced/ Separated	4	3 (75.0)	0	1 (25.0)	
Widow/Widower	5	4 (80.0)	1 (20.0)	0	
**Religion**	110				.632
Christianity	99	81 (81.8)	9 (9.1)	9 (9.1)	
Islam	11	8 (72.7)	1 (9.1)	2 (18.2)	
**Educational Level**	110				.497
JHS and Below	21	19 (90.5)	1 (4.8)	1 (4.8)	
Senior High/ O’/A ‘Level	36	29 (80.6)	2 (5.6)	5 (13.9)	
Tertiary	53	41 (77.4)	7 (13.2)	5 (9.4)	
**Occupation**	109				.399
Employed	56	43 (76.0)	6 (10.7)	7 (12.5)	
Unemployed	53	46 (86.8)	3 (5.7)	4 (7.5)	
**Reason for first facility visited**					.001
Previous patient	12	11 (91.7)	1 (8.3)	0	
Type of symptoms	35	30 (85.7)	4 (11.4)	1 (2.9)	
Recommendation	15	6 (40.0)	3 (20.0)	6 (40.0)	
Proximity	22	21 (95.5)	0	1 (4.5)	
Patient works at the facility	4	4 (100.0)	0	0	
Needed to be treated	19	15 (78.9)	1 (5.3)	3 (15.8)	

* p-values obtained from chi-square tests of association

#### Knowledge and perception

There was a statistically significant association between level of education and prior knowledge of their condition, *X*^2^ (2, *N* = 107) = 12.28, *p* = .002.

Knowledge characteristics of respondents relating to their condition indicated that only 21 participants (19.6%) had prior knowledge of their disease before they were diagnosed. This was distributed as follows: 8/21 (38.1%) knew that the condition was autoimmune in nature; 9/21 (42.9%) had prior knowledge of the symptoms associated with the conditions; 1/21 (4.8%) thought it was hereditary; 1/21 (4.8%) said that it was incurable; 2/21 participants (9.5%) had simply heard of the condition. Participants’ source of information varied with the majority learning about the condition through personal reading and media sources (health education programmes on radio and television). Fewer participants mentioned knowledge acquired through a personal contact with someone who has the condition or through family and friends.

Majority of participants (71%; 78/110) had no idea what caused their illness. The remaining participants gave multiple answers: 8/32 (25%) thought it might be due to a spiritual cause; environmental factors– 4/32 (12.5%); diet– 4/32 (12.5%); ageing– 3/32 (9.4%), with the rest attributing the cause to biological, including genetic causes 13/32 (40.6%) or stress-related factors 9/32 (28.1%).

Assessing the perception of others regarding their illness, 48/110 (43.3%) said they did not know what others thought about them. Among the remaining participants, the responses were sympathy, laziness, and a spiritual cause. They reported that others treated them differently or complained about them. The condition affected the participants in various ways: 34% (37/110) felt they were not accepted by the community; 25% (27/110) of the respondents said they felt embarrassed, 17.6% (19/110) reported that others thought less of them; 5.6% (6/110) said that others thought their condition was contagious, and 66.7% (73/110) were not affected by any of the stated factors.

Almost half of the respondents (49.5%) indicated that their condition impacted their ability to perform at school, work, or ability to find employment. These were due to symptoms namely, “weakness/tiredness”, “pain” and “deformities”. Twenty percent (20%) of the respondents reported that their illness affected their ability to form new relationships or find a marriage partner and 9.2% (10/110) concealed their diagnosis from a confidant or loved one.

#### Complementary and alternative medicine use—I-CAM-Q

Forty participants (36.4%) utilised certain complementary and alternative treatment and care options from a spiritual leader and a herbalist. It was also observed that once the participants were diagnosed in the rheumatology clinic and were receiving treatment, 18.2% still expressed the desire to seek alternative help. Praying for one’s own health as a form of self-help practice was recorded in 93% (37/40) of participants. See S1 and S2 Figs.

### Qualitative results

In-depth individual interviews (IDI) were conducted until data saturation and one focused group discussions (FGD) was done to supplement the data obtained from the individual interviews. The participant characteristics are presented in Table 3. Five main themes influencing health seeking behaviours emerged: Knowledge and Perceptions of ARDs; Care-seeking Behaviour; Social Reactions and Stigma; Negative Feelings; and Coping Strategies. In addition, the subthemes and quotes have been presented in S1 Table.

**Table 3:** Focus group discussion and in-depth interview participant characteristics.

Disease	Female	Male
Rheumatoid Arthritis	5	2
Systemic Lupus Erythematosus	9	1
Mixed connective tissue disease	2	1
Total	16	4

#### Knowledge and perception

Participants attributed their condition mostly to biological factors, lifestyle factors and spiritual causes. Those who attributed their condition to spiritual factors revealed their perception was shaped by the sudden, scary onset of symptoms which did not seem to be normal.

*“I thought it was spiritual because it was very strange*. *It is still there in my mind*. *I will not lie to you*, *but I also think it is my immune system*. *That is why I said bit of everything*. *I can’t leave the spiritual part out…” (FGD)*

Perception of symptoms influenced participants’ health-seeking behaviour, with certain symptoms being seen as more serious than others. In such cases, care seeking was quicker and more direct. The presence of swollen legs, rashes, and altered mental state, for example, were seen as an urgent problem requiring immediate attention. Other symptoms such as joint pain were attributed to less serious causes. Patients referred to it as “simple rheumatism” or related it to something they were more familiar with like bone pain arising from sickle cell disease. Patients who engaged in high level of physical activity related aches and pains to physical stress and injury of the joints, thus they sought formal help late, usually relying on self-medication.

*“I told you I have sickle cell disease and I already have bone problems so when it started I didn’t think of anything. I thought it was the normal crisis I get. When it was affecting me I will come to the SCD clinic and report. So through tests, I was eventually referred here”. (FGD)*

However, worsening symptoms, decline in physical health and recommendation from significant others pushed participants to seek help.

*“It was like malaria*, *when you go they give you the medicine then it will come back again so I stopped going to the hospital*. *I will go to the pharmacy to get the medicine that I know will work*, *but later on I said no*, *I have to go to the hospital*. *That is why I went to 37 Hospital*. *I was diagnosed with SLE and referred here” (FGD)*

#### Care-seeking behaviour

Perceptions about the origins of symptoms impacted the decisions about where care was initially sought. For those who attributed their illness to biomedical causes, they would more often seek formal healthcare options. In exploring participants’ choice of health care facilities, it was revealed that they relied heavily on pharmacists for analgesia and basic advice. There was evidence of multiple attendance to several facilities at a primary care level before escalating care to specialist rheumatology services. We further observed that interpretations of disease causality varied and participants moved between seeking biomedical, traditional and religious explanations for their symptoms in varying orders. Some participants described the parallel existence of spiritual, herbal and biomedical components to seek healing.

*“I have been to several hospitals*, *from polyclinics to big government hospitals*. *I have tried herbal treatment; I have been to the church for prayers*, *then went back to another herbal clinic*. *The last one I went to was a prayer camp because I believe in miracles before I finally came here (hospital)*.*” (IDI; Participant 1)*

Participants readily sought alternative systems of health care when one form of care was deemed ineffective or unable to provide a cure. This was observed frequently amongst participants who sought help first in the non-specialist medical centres, but failed to improve, so resorted to traditional and/or spiritual healers. The reverse was also noted.

*I visited several private hospitals which were of no help so my husband took me to a prayer camp*. *After*, *I visited Esukuma Government Hospital and they gave me drugs based on the symptoms I talked about but it wasn’t working so I decided to try herbal centres which were of no help too*. *After*, *I went to Apam Hospital and they referred me to Korle Bu*. *(IDI; Participant 6)*

#### Social reactions and negative feelings

It was reported that behaviour from others shaped perceptions, and consequently decisions regarding health seeking practices. Participants expressed concerns about how they were perceived and treated both by family and others. They reported that the negative interactions resulted in concealing their symptoms from close contacts, and in some cases not willing to seeking medical assistance. They also experienced challenges with emotional state, education, relationships and childbearing.

*“It affects me emotionally, sometimes I think about it. Where could this come from? What are people saying about me when they see me? Yes, I think about it.” (FGD)*

#### Coping strategies

Participants’ choice to use alternative means of seeking recovery was based on their knowledge of what caused their illness. They engaged in self-help practices, such as resting more, improving their diet and self-medicating as means of obtaining relief from their symptoms.

*“I rest*, *but sometimes I have to try hard and go on with my normal duties*, *even when I am sick*.*” (FDG)*

When participants gave account of their subjective well-being and how they were dealing with the illness, 8 out of 10 reported using prayer/spirituality and rituals as means of coping with their illness and due to the strong personal beliefs they had. They derived hope and comfort form the relationship they encountered with a higher being or God and relied on prayer encounters as a form of healing.

*“It hasn’t been long since this happened, but God has given me the courage. Since I have God I have a future.” (FGD)*

Social support from family and friends was also identified as being helpful in dealing with the challenges of the illness as expressed by participants.

“*My family have been so supportive I couldn’t have done it without them” (FGD)*

## Discussion

To our knowledge, this is the first study to document health seeking behaviour of Ghanaian patients with autoimmune rheumatic diseases. Results showed significant variation in the time taken for patients to seek health care; the type of facility they chose to seek care from; and the factors influencing these behaviours. Some patients were found to act relatively quickly once new symptoms began through varied approaches. These included rest from regular activities and self-medication with orthodox medicines/traditional home remedies. Other practices were attendance at an orthodox health facility, presenting at an herbal clinic, or engaging in prayers and rituals at the church/prayer camp. However, there was a significant variation among the patients, ranging from a few days up to a delay of several years. Although the use of over-the-counter medications and lifestyle adaptations are recognised causes for why patients may delay in seeking help from a healthcare practitioner, we recognised an additional delay factor owing to the use of herbal clinics and prayer camps. This is consistent with a study by de-Graft Aikins et al which reported a similar delay in receiving medical help among African societies [20]; this is in contrast to European studies which report a relatively shorter delay of up to 24 weeks [21, 22].

In our study, several factors contributed to the delay of 12 months from the time participants first sought help to obtaining the final diagnosis. The qualitative data highlighted challenges in accurately identifying signs and symptoms of ARDs by the patients; a significant gap in medical expertise to diagnose these illnesses which led to the participants attending different medical clinics, receiving variety of treatments and prolonging onward referral to specialist services. The patients’ choice to attend herbal centres and prayer camps/churches, either due to their personal belief in the supernatural cause of their illness or lack of appropriate, timely diagnosis and management from a medical centre was recognised as a delay factor. This differs from other Western studies showing that once patients are seen by a primary care physician, onward referral to specialist rheumatology services for diagnosis and management is usually within 3 months [21–24].

Knowledge of the condition prior to diagnosis was very limited and health-seeking behaviour was influenced in part by their perception of the disease and symptoms. We found that in patients who considered their symptoms as “non-serious”, care seeking was delayed, as has been reported by other studies [24, 25]. Those who perceived the cause of their illness to be supernatural sought treatment of a supernatural nature. It is known that cultural practices inform contextual knowledge, which in turn can influence health-seeking behaviour [26, 27]. In contrast to high income countries where orthodox medicine forms the main stay of treatment, in Ghana, traditional medicine receives more attention due to a function of culturally-mediated spiritual beliefs and the need for absolute cures [20, 28, 29]. In a study of chronic kidney disease patients. Kretchy et al observed that many participants believed that biomedical treatments alone could not manage their illness, so they continued to participate in customary healing practices, such as homeopathy, naturopathy and osteopathy [30].

In our study, the practice of healer shopping was noted among the participants, co-existing alongside orthodox care received from the specialist rheumatology centre. Participants routinely sought alternative care from the church/prayer camp and herbalist. Religiosity was valued highly among the participants and engagement with the spiritual community constituted one of the coping strategies. The I-CAM-Q tool clearly showed co-existence of these alternative practices and that praying for one’s own health was the most common self-help practice used by the participants.

We found that other factors influencing health-seeking behaviour were gainful employment, higher educational level and access to medical health facilities which were associated with earlier attendance to specialist rheumatology services. Lartey et al. reported that patients with a higher educational level had a better biomedical understanding of the disease and possessed more accurate information on health seeking, hence were more inclined to follow orthodox treatment pathways [31]. In other studies, those with higher income levels have enhanced access to formal healthcare [27], whereas those in the lower income bracket may be pushed towards traditional medicine, which is thought to be less costly [20, 30, 32]. In our study, proximity to a health facility was a significant determinant of choice of facility visited. The participants largely came from the capital region, Greater Accra, which has a huge saturation of medical clinics and hospitals [33]. Gergianaki et al. reported that patients from highly urbanised regions are at an advantage due to access to specialist facilities, better education and socio-economic status amongst other factors [34].

A strength of this study is that in adopting the mixed method study, the qualitative phase added further insight into the care-seeking behaviour and highlighted the various referral patterns. Participants visited several facilities at the primary care level before escalating care to specialist rheumatology services. They were also more open about their practice of medical pluralism, which was in contrast to findings from the survey reporting that the rheumatologist was mostly engaged as a second or third option. Secondly, the participants reported the negative impact and stigma the illnesses had on their lives and the effect it had on illness behaviour. For example, they concealed their symptoms from close contacts, which led to a delay or avoidance in seeking medical assistance. However, our study also had some limitations. Data was collected from a single specialist centre in an urban area suggesting that patients had an implied health-seeking practice biased towards orthodox medical care. An extension to rural areas using longitudinal study methods is recommended for future studies. Secondly, patient numbers were low, though this may reflect the low occurrence of the conditions or the problem of underdiagnosis or misdiagnosis. In addition, Religiosity and spirituality were noted to be key aspects to health seeking behaviour, hence validated tools to formally assess these variables and their impact on health-seeking behaviour should be used.

### Conclusion and recommendations

This study brings to the fore a huge gap that exists in the knowledge and perception of ARDs among Ghanaian patients. A multiple health-seeking medical culture was observed and can be attributed to parallel biomedical and spiritual causal theories, belief in traditional healing and unfulfilled healthcare experiences. These led to a delay in diagnosis of ARDs amongst Ghanaian patients. To effectively bridge this gap, awareness campaigns for the general public about the conditions are necessary. Clinicians and healthcare practitioners must become more involved in dispelling these negative attitudes and to be able to do this, they also require education and training about the conditions. Finally, strategic planning to integrate the biomedical and traditional care services is required. This multi-pronged approach will enable earlier presentation, accurate diagnosis of these rheumatological conditions and improved patient outcomes.

## Supporting information

S1 Fig Flow chart showing summary of participant recruitment into the quantitative and qualitative arms of the study. (TIFF)

S2 Fig Complementary services utilised—I-CAM-Q. (TIFF)

S3 Fig Self help practices—I-CAM-Q. (TIFF)

S1 Table Themes and examples of quotes from the thematic analysis. (PDF)

S1 Appendix Study questionnaires. (PDF)

S2 Appendix Interview guide. (PDF)

S3 Appendix Dataset. (SAV)

S4 Appendix COREQ checklist. (PDF)

S5 Appendix Transcripts from the interviews. (ZIP)
