# Supplementary figures and images for: Health-seeking behaviour, referral patterns and associated factors among patients with autoimmune rheumatic diseases in Ghana: A cross-sectional mixed method study
Source: PLoS One. 2022 Sep 12;17(9):e0271892. doi: 10.1371/journal.pone.0271892 (PMC9467363; doi:10.1371/journal.pone.0271892)

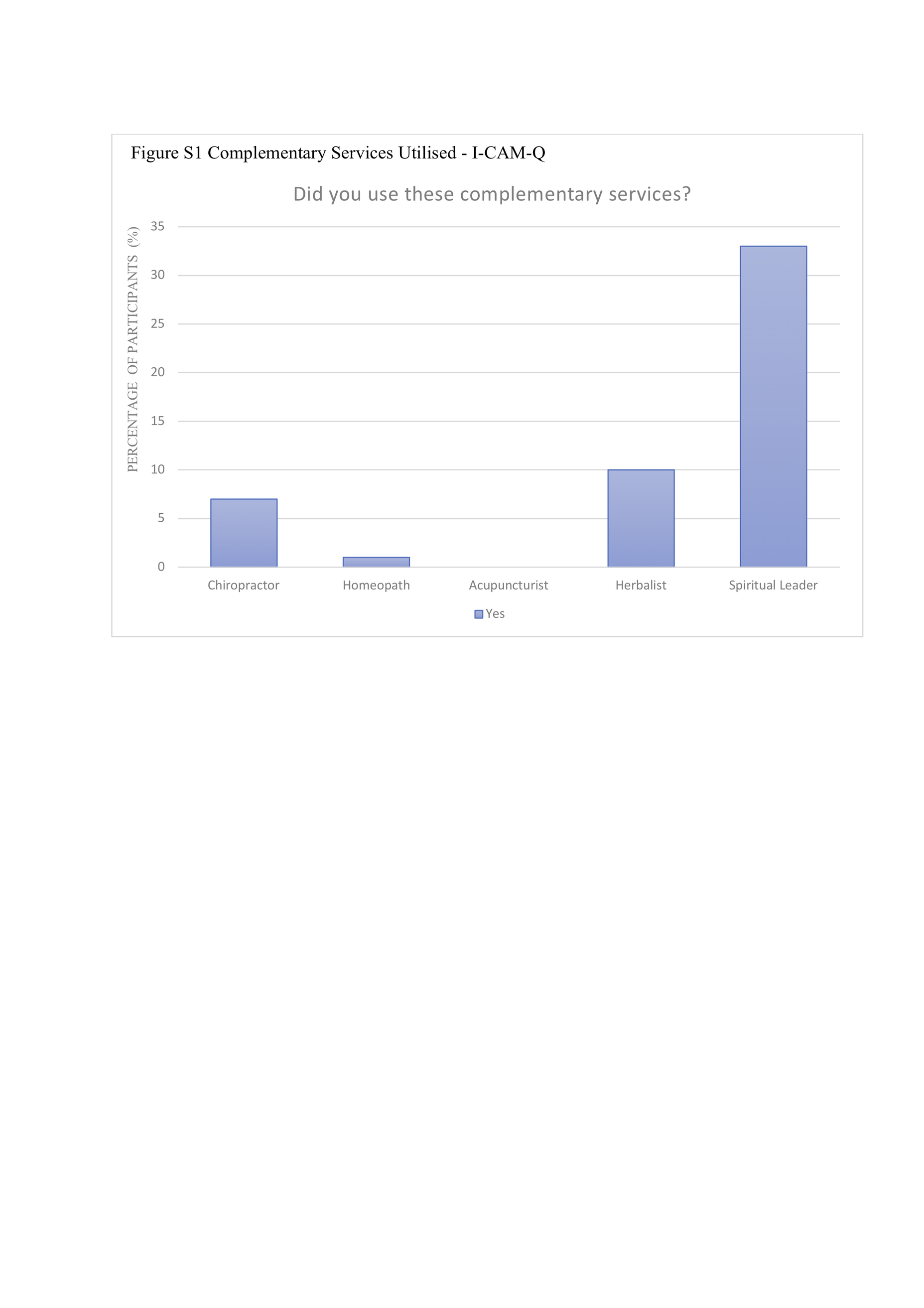

Supplement: S2 Fig — (TIFF) [file pone.0271892.s002.tiff]

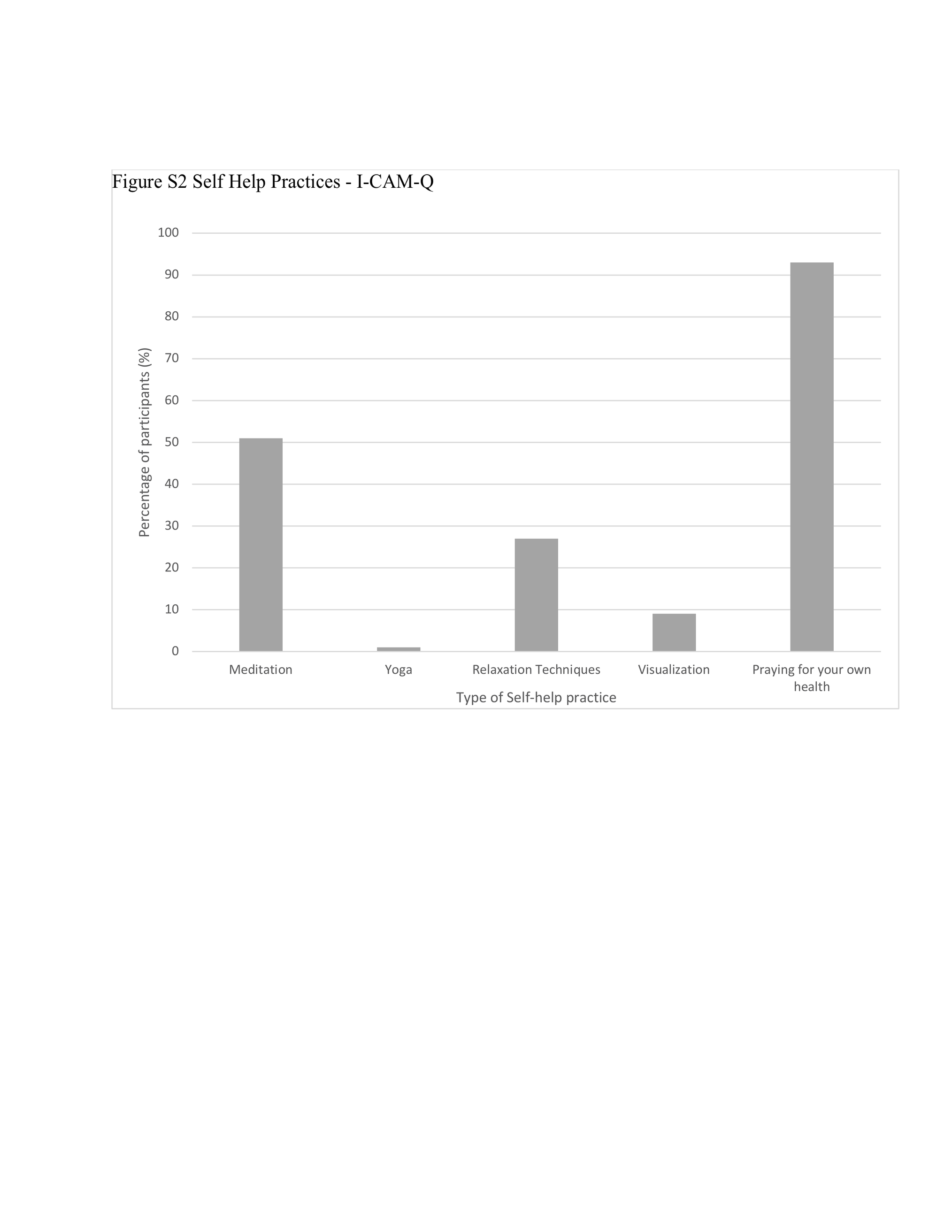

Supplement: S3 Fig — (TIFF) [file pone.0271892.s003.tiff]
