# Supplementary material for: Health-seeking behaviour, referral patterns and associated factors among patients with autoimmune rheumatic diseases in Ghana: A cross-sectional mixed method study
Source: PLoS One. 2022 Sep 12;17(9):e0271892. doi: 10.1371/journal.pone.0271892 (PMC9467363; doi:10.1371/journal.pone.0271892)
Supplement: S1 Table — (PDF) [file pone.0271892.s004.pdf]

S1 Table. Themes and examples of quotes from the thematic analysis

Main themes	Sub-themes	Quotations
Knowledge and perception about condition	Biological	I know it is about our immune system (*FGD)*
	Lifestyle	Lack of exercise and if you don't eat well *(FGD)*
	Spiritual	When the symptoms started I did not understand, the way the sickness is, you will think maybe "someone is causing it". (FGD)*[In local parlance this phrase refers to a spiritual curse]*
Care seeking:<br><br>Personal actions of relief	Rest or sleep	Because I want the pains I feel to reduce, I rest *(IDI; Participant 4, female)*<br><br>I rest so that I will not stress myself.
	Visit health professionals	I go to the pharmacy *(FGD)*
	Self-medication	I use first aid *(IDI; Participant 10, female)*
Care seeking:<br><br>Desperate actions for permanent treatment	Healer shopping	"My condition was not improving after taking herbal medicine" (FGD).<br><br>"…so many, King David, Atom, I can't mention all. They are many, more than 10 places. Even herbal hospitals, plenty…but later on I said no I have to go to the hospital. That is why I went to 37 Hospital where I was diagnosed with SLE, then I came here". (FGD)
Effects of condition:<br><br>Social Reactions	Relationships	I want to quit because of my condition. He does not treat me the way he is supposed to treat me. When he is supposed to help me he doesn't. When I ask for something he gets angry…*(IDI; Participant 6, female)*
	Education	**"**When I was sick I wasn't able to go to school; I was always at home feeling weak. I wasn't able to write exams. It has affected me" *(FGD)*
	Child bearing	"Yes, I am 32 and I have not given birth, that will push men away, maybe they will want a child." *(FGD)*
	Non-disclosure	Ooohh! Noo! If you don't know, you don't know. That's how it is. I am not that type of person who prefers to keep things, because if I tell you, there's nothing that you can do about it. So if you know, you know. If you

		don't know, that's all. *(IDI; Participant 9, female)*<br><br>"Initially people thought I had HIV because I was losing weight. I was scared to go to the hospital in case it was true." (FDG)
Effects of condition: Negative Feelings	Anxiety	Like sometimes when you want to do something, even when you work and you feel tired then it seems like you are feeling the sickness again, then you are in pain. Then it comes to mind, is it (illness) coming back. So I have to relax. *(IDI; Participant 5, female)*
	Pessimism	There are periods when I get very emotional. It is like the world is coming to an end. But you start getting guilty within yourself. It goes on and off. *(FGD)*
Coping strategies	Religious	It hasn't been long since this happened, but it's like God has given me the courage……Since I have God I have a future. *(FGD)*
	Significant others	My family have been so supportive I couldn't have done it without them *(FGD)*
	Knowledge of diagnosis	Knowing about it has helped me manage it well *(IDI; Participant 9, female)*
