# Supplementary material for: Health-seeking behaviour, referral patterns and associated factors among patients with autoimmune rheumatic diseases in Ghana: A cross-sectional mixed method study
Source: PLoS One. 2022 Sep 12;17(9):e0271892. doi: 10.1371/journal.pone.0271892 (PMC9467363; doi:10.1371/journal.pone.0271892)
Supplement: S1 Appendix — (PDF) [file pone.0271892.s005.pdf]

UNIVERSITY OF GHANA SCHOOL OF MEDICINE AND DENTISTRY

COLLEGE OF HEALTH SCIENCES

PARTICIPANT INFORMATION SHEET

STUDY TITLE: **Social determinants of health seeking behaviour and the impact on referral patterns and outcomes among Ghanaian patients with chronic inflammatory rheumatic diseases**

NAME OF INVESTIGATOR: Dr Maame-Boatemaa AMISSAH-ARTHUR

Address: Department of Medicine and Therapeutics,

University of Ghana School of Medicine and Dentistry,

KBTH

Telephone: 057 080 3449

Email: mbaarthur@doctors.net.uk

Invitation

You are being invited to take part in a research study. Before you decide, it is important for you to understand why the research is being done and what it will involve. Please take time to read the following information carefully. Talk to others about the study if you wish. Ask us if there is anything that is not clear or if you would like more information. If you do decide to take part, you are free to withdraw at any time without explanation and your subsequent treatment will not be affected. There will be no compensation for taking part in the study.

Why have I been chosen?

You have been invited to participate because you have been identified as having one of the listed conditions - rheumatoid arthritis (RA) or inflammatory arthritis, systemic lupus erythematosus (SLE/Lupus), mixed connective tissue disease (MCTD), systemic sclerosis (SSc), polymyositis/dermatomyositis (PM/DM) or undifferentiated connective tissue disease (uCTD) and are being looked after in the specialist rheumatology clinic or currently on admission and newly diagnosed with one of the above conditions.

Do I have to take part?

No. It is up to you to decide whether or not to take part. If you do, you will be given this information sheet to keep and be asked to sign a consent form. You are still free to withdraw at any time and without giving a reason. A decision to withdraw at any time, or a decision not to take part, will not affect the standard of care that you receive.

What is the purpose of the study?

The purpose of this study is to explore underlying factors which inform an individual's pattern of behaviour when it comes to seeking help for their autoimmune inflammatory rheumatic disease at the point of initial symptoms and subsequently during their long term management. Information from the study will allow clinicians further understand the reasons behind the advanced nature of autoimmune diseases at presentation and its associated morbidity and mortality and ultimately structure health care to improve clinical outcomes.

What will happen to me if I take part?

You will be requested to complete a questionnaire/interview which will provide information about yourself and your health status. Oral responses given during the interview will be recorded using a recording device, such as a tape recorder, to allow accurate capture of data and allow analysis to occur at a later time after the interview has taken place. There will be no risk to your health if you participate in this study.

All information gathered will be made available only to the investigators of the study to enable them to draw conclusions. All information will be handled with utmost confidentiality and will have no impact on your current or future health care within the facility.

Where can I obtain further information?

If you require further information or have specific questions relating to this study, please do not hesitate to ask the researchers who approach you or use the contact details above.

Thank you for taking time to read this information sheet and considering taking part in this study.

ID No:

CONSENT FORM

Study title: **Understanding social determinants of health seeking behaviour and the impact on referral patterns and outcomes among Ghanaian patients with chronic inflammatory rheumatic diseases**

Please initial box

1. I confirm that I have read and understand the information sheet for the above study. I have had the opportunity to consider the information, ask questions and have had these answered satisfactorily. ☐

2. I understand that my participation is voluntary and that I am free to withdraw at any time, without giving any reason, without my medical care or legal rights being affected. ☐

3. I understand that relevant sections of any of my medical notes and data collected during the study may be looked at by responsible individuals from The University Ghana School of Medicine and Dentistry, from regulatory authorities or from Korle Bu Teaching Hospital, where it is relevant to my taking part in this research. I give permission for these individuals to have access to my records. ☐

4. I agree to my general practitioner/doctor being informed if any health concerns arise and you need to seek urgent care. ☐

5. I agree to take part in the above study. ☐

Name of Patient	Date	Signature
____________________	________________	______________

Researcher	Date	Signature
____________________	________________	______________

ID no:

Questionnaire

Please read through and answer ALL questions. Tick the fields that apply.

ID number:

A.

1. Age:……………………….

2. Gender: 1 – Male [ ] 2 – Female [ ]

3. Ethnicity:

1 – Akan	[ ]	4 – Mole/Dagbani	[ ]
2 – Ga/Dangbe	[ ]	5 – Ghanaian other	[ ]
3 – Ewe	[ ]	6 – non Ghanaian	[ ]

4. Marital status:

1 – Married/co-habiting	[ ]	3 – divorced/ separated	[ ]
2 – single/never married	[ ]	4 – widow/widower	[ ]

5. Religion:

1 –Christianity	[ ]	2 – Islam	[ ]
3 - African traditional religion	[ ]		
4 – Other	[ ]	Please state:……………	

6. Highest educational level attained:

1 - No school	[ ]	4 - Senior high/O'/A' Level	[ ]
2 – Primary	[ ]	5 – Tertiary	[ ]
3 - Middle school/JHS/JSS	[ ]		

Number of years of Education:…………………………………………...

7. Where do you come from for clinical care:

1 – Greater Accra region	[ ]	6 – Volta region	[ ]
2 – Central region	[ ]	7 – Eastern region	[ ]
3 – Ashanti region	[ ]	8 – Western region	[ ]
4 – Northern region	[ ]	9 – Upper east region	[ ]
5 – Upper west region	[ ]	10 - Brong Ahafo region	[ ]

B. **ID no:**

8. Occupation:

1 - Professional/Technical/managerial [ ]

2 - Clerical/ secretarial [ ]

3 - Sales and services [ ]

4 - Skilled manual (eg, carpenter, mechanic…) [ ]

5 - Unskilled manual (labourer, cleaner…) [ ]

6 - Agriculture (farming, fishing) [ ]

7 – Student [ ]
8 – Unemployed [ ]

9 – Other [ ] Please specify………………………

9. How long have you been in employment?... (months)

10. Earnings per month in cedis (GHC):

1 – GHC < 500	[ ]	4 – GHC 2000 – 3999	[ ]
2 – GHC 500 – 999	[ ]	5 - GHC> 4000	[ ]
3 – GHC 1000 – 1999	[ ]		

Household:

11. How many people live in your home? ....

12. Are you the head of household? Yes [ ] No [ ]

13. If no, who is the head of household to you?

1 – Parent	[ ]	2 - Spouse/partner	[ ]
3 - Uncle/aunt	[ ]	4 – Sibling	[ ]
5 – Child	[ ]		
6 – Other	[ ]	Please specify………………	

14. Who decides on household spending, including healthcare?

1 – Self	[ ]	4 - Uncle/aunt	[ ]
2 – Parent	[ ]	5 – Sibling	[ ]
3 - Spouse/partner	[ ]	6 – Child	[ ]
7 – Other	[ ]	Please specify………………..…	

15. Can you or your sponsor afford your investigations and medication currently?

Yes [ ] No [ ]

C. **ID no:**

16. Diagnosis:

1 – Rheumatoid Arthritis	[ ]
2 – Inflammatory Arthritis	[ ]
3 – SLE/Lupus	[ ]
4 – Systemic Sclerosis/Scleroderma	[ ]
5 – Polymyositis	[ ]
6 – Dermatomyositis	[ ]
7 – Mixed connective tissue disease	[ ]
8 – Undifferentiated connective tissue disease	[ ]
9 – Primary Sjogren's Syndrome	[ ]
10- Systemic vasculitis	[ ]

17. Have you ever (previously) heard of your illness/condition prior to diagnosis?

Yes [ ] (Answer Q.18,19) No [ ]

18. What did you know about your current illness prior to diagnosis?

…………………………………………………………………………………………………

19. Where did you find out about the condition prior to diagnosis?

…………………………………………………………………………………………………

20. How did you find out about the condition prior to diagnosis?

…………………………………………………………………………………………………

21. Age at onset of symptoms:……… Age at diagnosis of illness:…………..

22. Initial symptoms: (tick as many as apply to you)

Morning stiffness	Raynauds phenomenon
Weight loss	Body/limb swelling
Rash/skin changes	limb/joint deformity(disability)
Joint pain	Depression
Joint swelling	Anxiety
Ulcers	Seizures
Fever	Fatigue/extreme tiredness
Hair loss	Chest pain
Body/muscle weakness	Shortness of breath

ID no:

D

23. How long did it take you from the time you started experiencing these initial symptoms to seeking help?..........................

24. Indicate the order of facilities visited from first to last:

1 – Local or non-specialist clinic [ ]

2 – Pharmacy [ ]

3 – District or regional hospital [ ]

4 - Tertiary Hospital (KBTH, KATH, TTH) [ ]

5 – Church [ ]

6 - Prayer camp [ ]

7 – Herbalist/Traditional healer [ ]

8 - Other Please specify……………………………

25. Total number of facilities visited………………………….

26. What were your experiences?

a. What informed your decision to choose the first facility?...

b. Did your condition improve? Yes [ ] No [ ]

c. Did you expect a cure? Yes [ ] No [ ]

d. Did you think you could get better without specialist help?

Yes [ ] No [ ]

e. Other, please explain…………………………………

27. Where was the final diagnosis made?..

28. How long was it from seeking help initially till the diagnosis was made?..

29. Was the diagnosis made at the same type of facility accessed at the start of the illness?

Yes [ ] No [ ]

30. If No, what influenced the change?

…………………………………………………………………………………
………………………………………………………………………...................

31. If Yes, why did you remain with this type of facility?

…………………………………………………………………………………

ID no:

32. What do you believe to be the basis of your illness?

…………………………………………………………………………………………
…………………………………………………………………………………………

33. What have you been told about your illness? ……………………………………………

a. By whom?..
b. Do you agree?..

34. What treatment have you been given?

Prednisolone	[ ]	Osteocare	[ ]
Hydroxychloroquine	[ ]	Folic Acid	[ ]
Methotrexate	[ ]	Nexium	[ ]
Azathioprine	[ ]	Omeprazole	[ ]
Mycophenolate mofetil	[ ]	Sulphasalazine	[ ]
Cyclophosphamide	[ ]	NSAIDs	[ ]

Other …………………………………………………………

35 Do you take it? Yes [ ] No [ ]

Reasons?...
....

36. Have you considered going somewhere else that offers different form of help once the diagnosis was made?

Yes [ ] No [ ]

If yes, state where……………………………………………………………………

Explain why?
....
....

37. Does your family support you in your decision to seek help for your condition?

Yes [ ] No [ ]

38. Do you have any other chronic health condition?

Yes [ ] No [ ]

If yes, please state……………………………………………………………..

E.

39. In your opinion, what do you think others perceive of you and your illness?

….……………………………………………………………………………………………
……………………………………………………………………………………………..

ID no:

40. Do any of the following affect you because of your illness? (Tick as many as apply to you)

1 - Feeling embarrassed [ ]

2 - Not accepted in a community [ ]

3 - Others think less of you [ ]

4 - Others avoid you [ ]

5- Others think your condition can be spread to others/contagious [ ]

6 – None of the above [ ]

41. Do you think the community discriminates against people with similar issues as you?

Yes [ ] No [ ]

42. Have you concealed your diagnosis from a confidant/loved one?

Yes [ ] No [ ]

If Yes, why? ……………………………………………………………

43. Do you think your illness will affect your ability to school, work/find employment?

Yes [ ] No [ ]

If Yes, explain further ……………………………………………………………

44. Do you think your illness will affect your ability:

a) To form intimate relationship/ find marriage partner? Yes [ ] No [ ]

b) To form new relationships in general? Yes [ ] No [ ]

If Yes, explain further ……………………………………………………………

Africentric Worldview Scale **ID no:**

Please answer the following question about how you go about your daily activities and your reaction to things around you. For each statement, indicate the level of agreement (or disagreement).

1. Strongly disagree (SD)

2. Disagree (D)

3. Neither agree nor disagree (N)

4. Agree (A)

5. Strongly agree (SA)

		SD	D	N	A	SA
1	I perform better on oral rather than written tasks.					
2	When greeting someone, I prefer verbal acknowledgements (rather than a nod or a hand wave).					
3	I feel that sometimes I do things “just because it feels, right.”					
4	I listen to my inner voice.					
5	I am likely to rely on my inner voice.					
6	I have to see something to believe it.					
7	I can tell when a close friend is in trouble					
8	Attending churches, mosques, or other places of worship is important to me					
9	I meditate and engage in other acts of faith.					
10	I believe in a spiritual force or power					
11	When stressed, I put my faith in a higher being.					
12	When I hear music I respond actively to it					
13	When speaking I am likely to use body language and hand gestures					
14	I view death as a spiritual event					
15	When things don’t work out, I try to see the positive side.					
16	People should be judged on who they are rather their material achievement					

17	It is expected that the elderly will be cared for by the younger generations.					
18	Older members of my family are relied on for advice /guidance					
19	It is not unusual for me to call close family friends “aunties, uncle, etc.”					
20	The ultimate value of a person is in his/her service to others					
21	My successful achievements are due to the support of significant others					
22	Remembering the past is as important as preparing for the future.					

NAFKAM International CAM Questionnaire (I-CAM-Q):
RECOMMENDED FOR USE IN STUDIES OF COMPLEMENTARY AND ALTERNATIVE MEDICINE (CAM) –Self-Administered Version

1. **Visiting health care providers**: Health problems may be attended to by a variety of complementary and conventional health care providers

Have you seen any of the following providers in the last 12 months?	Yes	No	Number of times you saw this provider in the last 3 months?	Please indicate the main reason you last saw the provider (tick only one)				How helpful was it for you to see this provider? (tick only one)			
				For an acute illness/condition, one that lasted less than one month	To treat a long-term health condition (one that lasted more than one month) or its symptoms	To improve well-being	Other (please specify the other reason)	Very	Somewhat	Not at all	Don't know
Physician											
Chiropractor											
Homeopath											
Acupuncturist											
Herbalist											
Spiritual leader											
Specified option: ……………...											
Other (please specify): ……………...											

ID no:

2. <u>**Complementary treatments received from physicians**</u> **(MDs)**

If you have <u>not</u> seen a physician in the past 12 months, please go to question 3.

Some physicians provide complementary, as well as conventional treatments

Have you received any of the following complementary treatments from a physician in the last 12 months?	Yes	No	Number of times you received this treatment in the last 3 months?	Please indicate the <u>main</u> reason you <u>last</u> received this treatment (check only one)				How helpful was it to receive treatment from the physician? (check only one)			
				For an acute illness/condition, one that lasted less than one month	To treat a long-term health condition (one that lasted more than one month) or its symptoms	To improve well-being	Other (please specify the other reason)	Very	Some what	Not at all	Don't know
Manipulation											
Homeopathy											
Acupuncture											
Herbs											
Spiritual healing											
Specified option: ……………....											
Other (please specify): ……………....											

ID no:

3. **<u>Use of Herbal Medicine and Dietary Supplements,</u>** including tablets, capsules and liquids.

For each category below please list up to three products you have used in the last 12 months.	Do you <u>currently</u> use this product?		Please indicate the <u>main</u> reason that applies to your <u>last</u> use (check only one)				How helpful did you find this product? (check only one)			
	Yes	No	For an acute illness/condition, one that lasted less than one month	To treat a long-term health condition (one that lasted more than one month) or its symptoms	To improve well-being	Other (please specify the other reason)	Very	Somewhat	Not at all	Don't know
**Herbs/Herbal Medicine**										
**Vitamins/Minerals**										
**Homeopathic remedies**										
**Other Supplements**										

Self Help Practices

Have you used any of the following self-help practices in the last 12 months?	Yes	No	Number of times you used this practices in the last 3 months?	Please indicate the main reason that applies to your last use of the self-help practice (check only one)				How helpful did you find this self-help practice? (check only one)			
				For an acute illness/condition, one that lasted less than one month	To treat a long-term health condition (one that lasted more than one month) or its symptoms	To improve well-being	Other (please specify the other reason)	Very	Somewhat	Not at all	Don't know
Meditation											
Yoga											
Qigong											
Tai Chi											
Relaxation techniques											
Visualization											
Attended traditional healing ceremony											
Praying for own health											
Specified option: ……………….											

ID no:
