# Supplementary material for: Health-seeking behaviour, referral patterns and associated factors among patients with autoimmune rheumatic diseases in Ghana: A cross-sectional mixed method study
Source: PLoS One. 2022 Sep 12;17(9):e0271892. doi: 10.1371/journal.pone.0271892 (PMC9467363; doi:10.1371/journal.pone.0271892)
Supplement: S2 Appendix — (PDF) [file pone.0271892.s006.pdf]

Interview Guide

Key areas to be explored:

- Knowledge
- Belief
- Attitudes/Practices
- Stigma
- General impact of condition on patient

1. What do you do when you're usually not well?
    a. Why that action was taken? What informed your decision
    b. Who determines where you go to? Personal decision or someone else determines for you?

2. About your current condition [before diagnosis]?
    a. Had you heard about your condition?[before you developed symptoms]{assessing knowledge in the public space}
    b. What did you know about it before, what were you told and by whom
    c. What do you think caused your problems [belief of causes – ageing process/wear & tear arthritis/ something I've done/work/lifestyle/spiritual cause/curse]
    d. Where did you first go to when the symptoms of this specific condition began?
    e. How long did it take you to go to this facility? [duration of onset of symptoms to seeking help]
    f. What prompted you to go there? [sudden vs indolent onset/presence or absence of physical signs/ symptoms interrupting daily activities, work, personal life/symptoms unusual and unable to explain]
    g. Did you visit any other facilities [herbal/traditional/prayer/church] and why?

3. About your condition [after diagnosis]?
    a. What do you understand now about your condition?
    b. What do you think is causing your condition [belief of causes – ageing process/wear & tear arthritis/ something I've done/work/lifestyle/spiritual cause/curse/other]
    c. After diagnosis have you felt the need to go to other facilities for further treatment and why? [alternative healthcare providers-PT/chiropracter/homeopath/acupuncturist/herbal/traditional/prayer/church
    d. Do you always take your medication as prescribed by the specialist? [yes/no why]
    e. Apart from prescribed medication from the specialist centre do you use any self-help practices to reduce your symptoms or achieve total healing – [herbal/prayers/food supplements/massage/manipulation therapy]

4. Who have you told about your condition? [family members/friends/work & school colleagues]

   a. How do they treat you knowing your condition [family members/friends/work & school colleagues]
   b. How do others [not informed individuals] treat you [in relation to visible clinical signs/physical body changes]?

5. How has your condition affected you and your ability to do things – physically, mentally, emotionally, socially?]
