# Supplementary material for: Health-seeking behaviour, referral patterns and associated factors among patients with autoimmune rheumatic diseases in Ghana: A cross-sectional mixed method study
Source: PLoS One. 2022 Sep 12;17(9):e0271892. doi: 10.1371/journal.pone.0271892 (PMC9467363; doi:10.1371/journal.pone.0271892)
Supplement: S4 Appendix — (PDF) [file pone.0271892.s008.pdf]

Table 1
Consolidated criteria for reporting qualitative studies (COREQ): 32-item checklist

No	Item	Guide questions/description	
**Domain 1: Research team and reflexivity**			
Personal Characteristics			
1.	Interviewer/facilitator	Which author/s conducted the interview or focus group?	Anna Gyaban-Mensah
2.	Credentials	What were the researcher's credentials? *E.g. PhD, MD*	MPhil Clinical Psychology
3.	Occupation	What was their occupation at the time of the study?	Clinical Psychologist
4.	Gender	Was the researcher male or female?	Female
5.	Experience and training	What experience or training did the researcher have?	She has been working on various health related research projects in the areas of CKD, autoimmune rheumatic diseases, HIV, and diabetes among others for the past 5 years. She is the editorial assistant

No	Item	Guide questions/description	
			for the Ghana International Journal of Mental Health.
Relationship with participants			
6.	Relationship established	Was a relationship established prior to study commencement?	Yes. A relationship was established in the first phase of the study.
7.	Participant knowledge of the interviewer	What did the participants know about the researcher? e.*g. personal goals, reasons for doing the research*	Participants were aware that their responses was to help identify gaps in referral patterns to inform clinical practice
8.	Interviewer characteristics	What characteristics were reported about the interviewer/facilitator? e.g. *Bias, assumptions, reasons and interests in the research topic*	The interviewer, based on previously established relationship with participants, provided a conducive and trusting environment for participants to share their experiences.
**Domain 2: study design**			
Theoretical framework			

No	Item	Guide questions/description	
9.	Methodological orientation and Theory	What methodological orientation was stated to underpin the study? *e.g. grounded theory, discourse analysis, ethnography, phenomenology, content analysis*	Thematic analysis<br>Page 6, Line 27-33
Participant selection			
10.	Sampling	How were participants selected? *e.g. purposive, convenience, consecutive, snowball*	Consecutive sampling based on participants' availability and willingness to participate in the second phase of the study<br>Page5, Line13-18
11.	Method of approach	How were participants approached? e.*g. face-to-face, telephone, mail, email*	Participants were called via telephone after they had completed the quantitative phase of the study<br>Page5, Line13-18
12.	Sample size	How many participants were in the study?	20 Participants<br>Page5, Line13-18
13.	Non-participation	How many people refused to participate or dropped out? Reasons?	10 people could not participate in the interviews due to lack of availability and distance to clinic

No	Item	Guide questions/description	
Setting			
14.	Setting of data collection	Where was the data collected? e*.g. home, clinic, workplace*	Outpatient Clinic<br>Page 4, Line 40
15.	Presence of non-participants	Was anyone else present besides the participants and researchers?	Yes, research assistants
16.	Description of sample	What are the important characteristics of the sample? *e.g. demographic data, date*	The sample consisted of patients with AIRDs who had consented and took part in the first phase of the study
Data collection			
17.	Interview guide	Were questions, prompts, guides provided by the authors? Was it pilot tested?	Interview guides was made up of open ended questions with prompts and follow up questions. The guide was pretested and amended before use<br>Page 6, Line 6-15
18.	Repeat interviews	Were repeat interviews carried out? If yes, how many?	No
19.	Audio/visual recording	Did the research use audio or visual recording to collect the data?	The data was audio recorded with the consent of participants

No	Item	Guide questions/description	
			Page 5, Line 10-12<br>Page 6, Line 14-15
20.	Field notes	Were field notes made during and/or after the interview or focus group?	Field notes were made after the interviews and focus group
21.	Duration	What was the duration of the interviews or focus group?	The interviews were mostly between 20- 30 minutes and the focused group discussion lasted for 45 minutes
22.	Data saturation	Was data saturation discussed?	Yes
23.	Transcripts returned	Were transcripts returned to participants for comment and/or correction?	No
**Domain 3: analysis and findings**			
Data analysis			
24.	Number of data coders	How many data coders coded the data?	Two
25.	Description of the coding tree	Did authors provide a description of the coding tree?	No

No	Item	Guide questions/description	
26.	Derivation of themes	Were themes identified in advance or derived from the data?	Themes were identified in advance
27.	Software	What software, if applicable, was used to manage the data?	Not applicable
28.	Participant checking	Did participants provide feedback on the findings?	No
Reporting			
29.	Quotations presented	Were participant quotations presented to illustrate the themes / findings? Was each quotation identified? e*.g. participant number*	Participants' quotations were presented to illustrate the themes and findings. Each quote was identified by the participant number and gender
30.	Data and findings consistent	Was there consistency between the data presented and the findings?	There was consistency between the data presented and the findings.
31.	Clarity of major themes	Were major themes clearly presented in the findings?	Major themes were clearly presented in the findings<br>Page 8, Line 30-34<br>Table

No	Item	Guide questions/description	
32.	Clarity of minor themes	Is there a description of diverse cases or discussion of minor themes?	Minor themes were adequately discussed<br>Page 8, Line 30-34<br>Table 3
