# Supplementary material for: Health-seeking behaviour, referral patterns and associated factors among patients with autoimmune rheumatic diseases in Ghana: A cross-sectional mixed method study
Source: PLoS One. 2022 Sep 12;17(9):e0271892. doi: 10.1371/journal.pone.0271892 (PMC9467363; doi:10.1371/journal.pone.0271892)
Supplement: S5 Appendix — (ZIP) [file pone.0271892.s009.zip › AUDIO 29.pdf]

**AUDIO 29**

**INTERVIEWER**: Please what do you do when you are usually not feeling well? What actions do you take?

**PARTICIPANT**: Errm if am not feeling well, the first thing is to call [redacted]. " *laughs*". I do hospital. Yes

**INTERVIEWER**: Why do you take that action?

**PARTICIPANT**: That's the only one that's proven to work, because I do not have any other option. If I do not do hospital, what will I do? Staying at home means you are still at home with the same condition, it hasn't changed. So call the doctor, get help yeah.

**INTERVIEWER**: So who decides on that decision yourself or a family member?

**PARTICIPANT**: Myself, no no myself.

**INTERVIEWER**: Errm… so before the diagnosis did you hear about your condition? Did you have any information?

**PARTICIPANT**: I didn't have information. I had heard of someone who had it but I just knew it was very rare and even that person used to go outside for treatment. The person wasn't doing treatment locally ahaaa.

**INTERVIEWER**: So what did you hear about it?

**PARTICIPANT**: Ohh.. In fact I didn't know the details but I only knew that it was terrible, there was no cure ahaaa.

**INTERVIEWER**: Did you know the person personally?

**PARTICIPANT**: No not personally, it was a friend's spouse so I don't know.

**INTERVIEWER**: So please before the diagnosis, what do you think caused your condition? Do you have any belief towards the condition?

**PARTICIPANT**: Hmm so my journey to diagnosis was very short. Very very short so I didn't have time to think about the causes. Yes it was very short.

**INTERVIEWER**: Please where did you go first when the symptoms began?

**PARTICIPANT**: I didn't have any symptoms. I was pregnant and I had proteins showing in my urine and my BP was high so the doctor felt it was pre-eclampsia so that's what he treating me for till I think I errm , I had crisis. I was just growing weak. They claim I was going into heart failure or something but then, and then that was how come I was transferred to Korle-Bu and

luckily, I got someone who insisted that [redacted] comes to take a look at me ahaa..

**INTERVIEWER**: So after the diagnosis, what do you understand about the condition?

**PARTICIPANT**: Ohh so I understand that it can be managed even though its unpredictable but there are a lot of people who are having normal lives with the condition so follow what your doctor says. Pray.

**INTERVIEWER**: So do you have any information as to what the condition is about?

**PARTICIPANT**: Yes yes so now I understand that it is an auto-immune thing. My immune system is gone hyper and then it's attacking my kidney in my case yes. It comes with other effects and it has other symptoms but luckily I don't have those ones so yhh…

**INTERVIEWER**: So where did you receive majority of the information?

**PARTICIPANT**: Errm I think that maybe errm internet and then the resolute page, the tri page. Am on the tri-page, the whatsapp group so I get to hear about people and they also put information there as well.

**INTERVIEWER**: So errm so after diagnosis, do you have the need to visit other facilities?

**PARTICIPANT**: No I don't.

**INTERVIEWER**: Why?

**PARTICIPANT**: I don't have any other condition that requires it, so it's just my medication and am fine.

**PARTICIPANT**: If you say other facilities like?

**INTERVIEWER**: Other hospitals, the need to try other hospitals for additional….

**PARTICIPANT**: No no no not at all.

**INTERVIEWER**: So how do you feel about the outcome? With your medication, the treatment here..

**PARTICIPANT**: Am perfect.

**INTERVIEWER**: When you say perfect, what do you mean?

**PARTICIPANT**: Am fine, like health wise am fine. My labs are clean, they are okay and anytime I have an issue, it's not difficult getting to anyone in [redacted]. [redacted] is accessible, [redacted] and if I have any issue I want to talk, I can call [redacted], I can call [redacted] about my appointment… so am fine.

**INTERVIEWER**: So do you always take your medication as prescribed?

**PARTICIPANT**: Oh yes

**INTERVIEWER**: Why

**PARTICIPANT**: *"laughs"* I don't want to come and sleep on the third floor, that's one motivation that keeps me and mine too was very aggressive because within a short while, I was like gone bad bad bad. So as much as possible, I don't want to go back.

**INTERVIEWER**: Oh okay. So apart from the prescribed medication, did you use any self-help practices maybe massage, other therapies, food supplements?

**PARTICIPANT**: No, okay yh I pray. I pray a lot. I am a Christian. I pray a lot. But apart from that, I don't do anything extra.

**INTERVIEWER**: Please have you told anybody about your condition?

**PARTICIPANT**: Yes. I told my boss's at work and then I think a few friends, those who are close to me know. It's not something that generally, I would meet you and be telling you about it no.

**INTERVIEWER**: So your family members ….

**PARTICIPANT**: Not even all my family members. Maybe those who knew when I delivered and I was going through the health crisis and they were visiting me yes, maybe they would know but after that, I don't discuss like, am going to the hospital, am on medication. I don't do that.

**INTERVIEWER**: So your husband knows…

**PARTICIPANT**: Oh yes he knows

**INTERVIEWER**: So how has the relationship being? Those who know about it, how do they relate? Anything different, any support?

**PARTICIPANT**: Ohhh well, my family is supportive like yh my family is very supportive so the treatment is the same. I don't get any special cheat even at work.

**INTERVIEWER**: so has it affected the relationship?

**PARTICIPANT**: No! We don't even talk about it actually. Like it doesn't come up for conversation.

**INTERVIEWER**: So has your condition affected you physically in your ability to do things?

**PARTICIPANT**: Physically, it hasn't, it hasn't.

**INTERVIEWER**: But emotionally, do you feel bad about it, think about it?

**PARTICIPANT**: I don't think about it. The only time I think about it is when my appointment, my review date has come. That's the only time I think about it *"laughs slightly"* but generally no.

**INTERVIEWER**: But socially?

**PARTICIPANT**: No no no.

**INTERVIEWER**: So in terms of coping, how do you cope?

**PARTICIPANT**: Errmm how do I cope? I don't stress. What I can do, I do. What I can't do, I find ways around it. So I do my own stuff but if for some reason this week I feel extremely tired, I will carry my things to the laundry, find someone to cook for me and then I will just rest. Well, of course with my kids I can't really rest but at least I take out that part, that physical exertion part and then just take it easy.

**INTERVIEWER**: So do you have hope? Hope that things will get better?

**PARTICIPANT**: Ohh from where I used to be and where I am now, I don't even think, I don't think about it. Am fine, for me am fine.

**INTERVIEWER**: Thank you very much

**PARTICIPANT**: You are welcome
