# Supplementary material for: Health-seeking behaviour, referral patterns and associated factors among patients with autoimmune rheumatic diseases in Ghana: A cross-sectional mixed method study
Source: PLoS One. 2022 Sep 12;17(9):e0271892. doi: 10.1371/journal.pone.0271892 (PMC9467363; doi:10.1371/journal.pone.0271892)
Supplement: S5 Appendix — (ZIP) [file pone.0271892.s009.zip › AUDIO 28 copy.pdf]

AUDIO 28

INTERVIEWER: please I will be asking you some set of questions.

PARTICIPANT: okay

INTERVIEWER: What do you do when u are usually not feeling well, on a normal day?

PARTICIPANT: aside my condition?

INTERVIEWER: yes with your condition or when you are not feeling well?

PARTICIPANT: ohhh… I take it normal and maybe like rest. If I have headache I take paracetamol and normal drugs so that I will be fine

INTERVIEWER: please why do you take that decision, either rest or paracetamol?

PARTICIPANT: if I have headache definitely I have to take something to calm the headache so I do take, that is the normal routine drug that we normal prescribe for us. So I just take the paracetamol then I rest

INTERVIEWER: so who determines that decision do you decide yourself or someone else?

PARTICIPANT: yes myself

INTERVIEWER: so about your current condition, before you were diagnosed have you heard, please what is your condition

PARTICIPANT: SLE

INTERVIEWER: before THE diagnosed have you heard ABOUT IT anywhere?

PARTICIPANT: I HAVE NEVER HEARD ABOUT IT

INTERVIEWER: SO WHAT DO THINK MIGHT BE THE CAUSE OF YOUR CONDITION?

PARTICIPANT: WELL AS I GOT TO KNOW THAE CONDITION, for me I think maybe it was stress because I think I was stressing myself within that time frame I was diagnosed of the condition

INTERVIEWER: what were you stressing yourself about ?

PARTICIPANT: hmmmm, my work. I was put on night shift and I was schooling too

INTERVIEWER: you are a [redacted]?

PARTICIPANT: yes, and I [redacted]. Hmm, so no leave. From [redacted] I have to come back to [redacted]. I think the stress was too much

INTERVIEWER: so you were diagnosed around that time

PARTICIPANT: mmmm yes. Yeah when I started [redacted]

INTERVIEWER: so where did you go when the symptoms started?

PARTICIPANT: I went to [redacted]. I was feeling pains in my joints like some fever. I couldn't sleep or stretch legs. I went to a medical assistant at usher [redacted] and he was like eerrrh fever so he prescribed diclo and some drugs for me but still. So me myself I told my in charge and my in charge wrote the referral letter for me that I should come to korle bu to see a physician

INTERVIEWER: what polyclinic?

PARTICIPANT: [redacted] polyclinic

INTERVIEWER: where is it?

PARTICIPANT: [redacted]

INTERVIEWER: so how long did it take between the onset of the symptoms and you visiting the polyclinic?

PARTICIPANT: I think within one month, I didn't keep long kraa

INTERVIEWER: okay, so what made you to go there?

PARTICIPANT: even I I was just like googling like online to the symptoms that I was having. So some say it is like HIV or (laughed). So I even checked for HIV but it was negative and I was like eeeiii, what is wrong with me? hmmmmm so I had to come

INTERVIEWER: so did you visit any other clinic maybe herbal, maybe other hospitals

PARTICIPANT: no, no please. From [redacted] straight to korle bu

INTERVIEWER: so now that you have been diagnosed have you had any understanding of what the condition is

PARTICIPANT: YES, yes please

INTERVIEWER: so can you share?

PARTICIPANT: the symptoms that I was having are the same things that I was told by my doctor. The symptoms that when you have the condition you will feel. And then I when I came the first time they made me do some labs. So when the labs came they explained everything to me and I understood it perfectly well.

INTERVIEWER: do you understand your condition

PARTICIPANT: yes

INTERVIEWER: can you share it?
PARTICIPANT: it is an autoimmune disease and it is like the immune system is fighting against

itself and then can also cause damage to the especially the organs like the kidney and then the liver. Joint pains too

INTERVIEWER: so where did you receive majority of your information from?

PARTICIPANT: from here, korle bu here. The first time i came I met one doctor so he wrote the lab for me. So when I brought the labs I was asked to see [redacted] . So it was there that [redacted] too explained

INTERVIEWER: so now that you have an information what do you now think is the actual cause? Do you think it is the stress? Do you think somebody is doing you? Do you think it is more of a biological cause? So now that you have been diagnosed and people, health professionals have explained thing to you, what do you thing yourself is casing it

PARTICIPANT: Well beginning, in the beginning as for human beings we will be thinking otherwise whether it is spiritual or here is this thing coming from. Because like am just there I have not done anything so what at all is this sickness coming from. So that thought also came to my mind and then with the labs and everything, I realized that it may be a family member of mine had but I don't know. So it more or less like the biological than the spiritual

INTERVIEWER: so what was you experience in polyclinic, how was your experience there

PARTICIPANT: oh it was cool but just that I think that place I don't think they even know about this condition so at they have to let me do some further labs too but because they don't know about it they just diagnosed me with fever and just gave me drugs

INTERVIEWER: SO AFTER BEING Diagnosed here, do you feel the need to visit other facilities?

PARTICIPANT: no because I have been coming here

INTERVIEWER: why please? Why don't you think you want to visit other places?

PARTICIPANT: to like confirm?

INTERVIEWER: no you want to visit other places for treatment?

PARTICIPANT: oh if there is a doctor, if a doctor is there who checks like SLE clients the patients why not? If I am referred there I will go. But since I will don't know of any clinic with such doctors it's here that I have been coming

INTERVIEWER: what about prayer camps, traditional, have you felt the need of going there maybe for spiritual

PARTICIPANT: I don't go there but I do pray on my own or like at church when we have prayer time or yes we go and I pray about it

INTERVIEWER: so how do you compare you current treatment to your previous treatment

PARTICIPANT: when they started giving me treatment. [redacted]?

INTERVIEWER: yes, your treatment here and then the one at [redacted]

PARTICIPANT: oh as for the [redacted] it didn't help me

INTERVIEWER: so right now that you have been diagnosed you are coming here for treatment, you come for review, how do you feel about the outcome?

PARTICIPANT: ooo its better, because the symptoms like I wasn't able to walk, when I get up from bed my legs will be stiff so it will take time for me to even stretch then I will be feeling feverish but I think even when I came and they gave me my drugs within two weeks like all the symptoms the swollen limbs everything have subside so its better

INTERVIEWER: do you always take your medicine as prescribed by the specialist

PARTICIPANT: mmmm

INTERVIEWER: why do you take the medication?

PARTICIPANT: if I don't take my condition will be worse so I have to take it. It, at times it's difficult when like you get up and you eat and like you have to take your medication but you have to

INTERVIEWER: so apart from your prescribed medication from you specialist do you use any self-help practices to reduce your symptoms. Maybe like food supplement, herbal medication or even go for some therapy

PARTICIPANT: maybe once in a while this herbal, maybe black seed I just take some but I don't take any traditional herbal

INTERVIEWER: so who knows about your condition? Family? Friends?

PARTICIPANT: my brothers they know about my condition

INTERVIEWER: you parents?

PARTICIPANT: as for my parents I haven't told them

INTERVIEWER: why?

PARTICIPANT: they will also be thinking about it. So it's my brothers that I told

INTERVIEWER: so how do they relate to you now that they know your condition?

PARTICIPANT: they have been asking me how I have been faring all the time

INTERVIEWER: have there been any difference in how they treat you?

PARTICIPANT: oh no, normal. We call each other and they ask how I am doing

INTERVIEWER: what about your friends

PARTICIPANT: I don't think I have told any of my friends

INTERVIEWER: are you in a relationship

PARTICIPANT: yes

INTERVIEWER: does the person know about it

PARTICIPANT: yes

INTERVIEWER: and how has he been relating to you so far?

PARTICIPANT: eeeerrrm well he says you need to stop the drugs

INTERVIEWER: why?

PARTICIPANT: I don't know. May be they are spiritual

INTERVIEWER: he believes it is spiritual?

PARTICIPANT: not like spiritual, but may be like God can heal me so I should have that faith and then stop taking the drugs and continue praying I will be fine

INTERVIEWER: and have you been thinking about that?

PARTICIPANT: oh me when he says it I also explain it to him. There was a time I think I didn't take the drug it got finished when I went I didn't get so two day later that I was asked to come and then… and those two days I really suffered. And I was like eeii see o the pains even you are telling me that faith I should stop but look at what has happening now so me I take it. I don't even let him see that I am taking it

INTERVIEWER: do you think he is been supportive?

PARTICIPANT: ooh in the beginning he was always asking have you taken your drugs. I don't know why as time went on he prevents me

INTERVIEWER: does it make you feel bad?

PARTICIPANT: no

INTERVIEWER: so how has your condition affected your ability to do things like physically?

PARTICIPANT: the beginning when I do something I easily get tired and maybe kneeling down then my knee. So they told me to, they prescribed calcium for me. But some once in a while I feel very tired so I don't do any stressful

INTERVIEWER: what about emotionally? Does it really affect does it make you feel sad

PARTICIPANT: oh yes, especially when I am in the house and I finish eating and I have to take my medication when I remove it and put it in my hands sometimes I cry “oh God why this thing”, but I take

INTERVIEWER: what about socially maybe hanging out with friends, has it affected you?

PARTICIPANT: oh no I do go out. Just that I don’t normally like afternoon I don’t like going because of the sun I have to maybe carry an umbrella and people will say “eeii this girl this afternoon carrying umbrella “ but evening, morning I do go out

INTERVIEWER: how do you deal with all these symptoms, what helps you to cope?

PARTICIPANT: I think, mmmmm when sometimes happen for me I do kind of sing. Yeah may be I will sing some inspirational songs, hymns. When I even forget something kraa like I will sit down sing and I will remember so those things I do sing to like encourage myself, yes.

INTERVIEWER: so how do you see your future? Because right now we don’t have a cure. Getting married in future one day, having children one day, do you thing about those things?

PARTICIPANT: yes I do, I do at times kraa, and will I die right now? Will i? Can I give birth? Do this? I do think about all those things

INTERVIEWER: and do you have hope that everything will get better?

PARTICIPANT: oh yeah I do have hope. Because. Since the way it started and then now that I don’t feel anything when I do the labs then I bring it too its ok. I think maybe I came earlier. So when the labs comes say kidney is fine, liver is fine so I don’t have any worry. When I take the drugs all that I do is I pray over it that “God I am taking the drug but don’t let it affect maybe my kidney this and this “I will be ok. I have hope but we will get a medications too then a cure for it.
