# Supplementary material for: Health-seeking behaviour, referral patterns and associated factors among patients with autoimmune rheumatic diseases in Ghana: A cross-sectional mixed method study
Source: PLoS One. 2022 Sep 12;17(9):e0271892. doi: 10.1371/journal.pone.0271892 (PMC9467363; doi:10.1371/journal.pone.0271892)
Supplement: S5 Appendix — (ZIP) [file pone.0271892.s009.zip › AUDIO TRANSCRIPTION.pdf]

AUDIO TRANSCRIPTION

QUESTION 1

Interviewer: what do you do when you are usually not well?

Participant 1: I sleep

Participant 2: I rest and also take medication because i am too quick to take a medication like pain killer

Participant 3: I rest but sometimes I have to go on with my normal duties. Sometimes I try hard even if I am sick I go on with my normal duties.

Participant 4: Go and see the doctor.

Participant 5: me sometimes I go for medications before I go and see the doctor. I go to the pharmacy

Participant 6: I take some medications

Probing Question: do you go to the pharmacy or you buy it on your own?

Participant 6: no, I take the ones I was given at the hospital

Probing Question: but normally what do you do?

Participant 6: I don’t go anywhere, I just get some medication at the pharmacy

Participant 7:Me in particular I am a [redacted] so I go to the [redacted].everything that happens to me I go there before they let me go where I want to go.

Participant 8: I don't normally fall sick. I was there one day when my hands. I was pounding fufu. When I realize my hands.

Participant 9: i also take paracetamol if I have headache but if it's a stomach ache I take it to the hospital

Participant 10: I take First aid.sickness is like headaches or stomachaches.I have first aid box in the house so I use it.I take the first aid one,two and the third time,I know I have to report to the hospital.but most at times I take it once and then I am OK.

Q1a

Interviewer: so why do you take the action you take, why do you sleep?

Participant 1: so that I will not stress myself

Participant 2: I don't want the pain to increase more that's why i take the medication.

Participant 3: with me I have to work for myself and cater for myself, so nothing can keep me down even sickness cannot keep me down

Participant 4: to get better

Interviewer: What informs that decision?

Participant 4: seeing the doctor I think is the best,

Participant 5: I go so that they give me some medications then I may be relieved, because when you feel the pains you have to be relieved from it

Participant 6: because of the pains I feel, I want it to reduce. That is why I came to the clinic

Interviewer: so you for instance, you sleeping you decide on your own to sleep or do other people advise you to sleep?

Participant 1: on my own

Participant 2: for me, on my own

Participant 3: yes that is my own decision

Participant 4: Like relatives or?

Probing Question: Yes yourself, relatives or friend?

Participant 4: Yes my dad he is the head, normally if you are not well he will say you should go to the hospital

Participant 5: my mum

Probing Question: Why does she?

Participant 5: sometimes I will go with her, other times she will go and get it for me

Participant 6: no one, I go on my own

Participant 9: I take it myself

Participant 10: Myself.

QUESTION 2

Interviewer: about your current illness have you heard about it before you were diagnosed?

Participant 1: no

Probing Question: nothing at all? No form knowledge about it?

Participant 1: no

Participant 2: I have heard about it, but i didn’t have so much knowledge about it

Participant 3: I didn’t know anything about it

Participant 4: it was once I was watching a program and the person had it and she was losing her hair. And that was the first time I heard about it

Participant 8: have not heard about it.

Participant 9: I haven’t had about it.

Participant 7: I have heard about it.I had about it at the clinic because normally I go to the clinic .I see people with such sickness come there.

Participant 10: I have heard about it before from friends I had from friends who have rheumatoid arthritis from their bones and Thing and the stigma that Comes with it.Me myself before I was referred here I was at [redacted] here. And there was a time I felt pains and I was what we called rheumatoid factor I was steam you could see I was steam,through medication I...

2b

Interviewer: what did you hear about it?

Participant 2: what I heard is that it is not curable, it can be managed but i didn’t know, I didn’t know, how will I explain it, I didn’t know too much

Participant 4: it was like the condition had to with, she was having joint pains also and she lost most of her because of that so that is what I got to know about the condition

Participant 5: no it was when I was diagnosed that I came to know about it

Participant 6: no I don’t know anything about it

Participant 7: they said is sickness of the bones.

Q2c

Interviewer: where did you hear about it?

Participant 2: on tv

Participant 4: America's next top model

Participant 10: Oh yes. Arthritis, Rheumatism, Yeah I heard it from friends and my friends to had some

Q2d

Interviewer: what do you think caused your problem?

Participant 1: I don't know what caused it

Participant 3: I don't know

Probing Question: you don't believe it is ageing or work or stress, lifestyle nothing?

Participant 1: no

Participant 2: me I thought two things; one, I thought it was hereditary and I thought it was my immune system being damaged

Probing Question: before you were diagnosed?

Participant 2: yes before

Participant 3: I didn't know what the cause was until recently that I read about it, some possible things could be the cause

Participant 5: the doctors said they don't know so me myself I don't know

Probing Question: Maybe your lifestyle?

Participant 5: sometimes they said it is your environment in which you live, sometimes depression but the actual cause they don’t know

Participant 6: I Heard it’s like someone, I don’t know how to describe it, it happens to someone who does not rest and does everything fast fast

Participant 10: aging,I don’t know if lack of exercise is Part, it could be a factor. Well you are the health people so I don’t know if diets affects it, probably.

Participant 7: What I know is if you don’t eat well, it can cause it.

Participant 8: The food we eat is part, now fertilizer and things are plenty.

Participant 9: the food that causes

Probing Question: don’t you think it’s spiritual?

Participant 6: no

Participant 5: first the way the sickness is you will think maybe someone is causing it. You will be going to all sort of places but then you will have to come to the hospital

Participant 4: no

Participant 7, 8, and 9: all laugh and said no

Participant 10: I doubt

Q2e

Interviewer: where did you go to when the symptoms started the first time?

Participant 1, 2 and 3: hospital

Participant 4: to the hospital

Participant 5: a lot

Probing question: can you mention some?

Participant 5: the one that i was referred from was 37

Probing question: before 37 where did you go?

Participant 5: [redacted], I can't mention all they are many, more than 10. Even herbal hospitals, plenty. It was like malaria, when you go they give you the medicine then it will come back again so i stopped going to the hospital. So the medicine that i know if I take I will be ok then I will go to the pharmacy but later on I said no I have to go to the hospital. That is why I went to [redacted] then I was diagnosed with SLE then i came here

Participant 6: I went to around about [redacted], afterward i came to [redacted] then they transferred me here

Probing question: did you visit a herbal hospital?

Participant 4,5 and 6: no

Participant 10: OK so I was at hematology. I was already diagnosed with something called ITP. it got to sometime I told the doctor I'm feeling pains my fingers all my neck and then he made me do a test about rheumatoid factor and it showed positive and there I was referred.it started with my fingers then my joints and here and here then my waist.

Participant 9: As for me my neck and my....so I went to ridge But they didn't see anything so they gave me painkillers and I drank so from there I went to [redacted] That time my here my hands and inside my legs so we went to do lab and it showed.

Participant 7: ass for me you see I told you I'm a sickle cell and I already have bone problems(sicknesses)so when it's started I didn't think of anything. I thought it was the normal crisis I get then it was doing me I will come and then I will report so through test, i was referred here.

Participant 6: me too I went to the hospital (cathedral) and they told me. i was done pounding fufu, I was just dressing it then my hand, then I felt pains here that's all.so I went to do lab then they transferred me to korlebu.

Q2f

Interviewer: how long did it take you to go to this facility you mentioned?

Participant 1: I don’t remember

Interviewer: can you tell me in months or years?

Participant 1: when it started I was going frequently sometimes the symptoms comes and goes

Interviewer: so when it started it didn’t take so long

Participant 1: no

Participant 2: for me six months but before that six months i was taking pain killers

Interviewer: so you will say the first place you went to was the pharmacy?

Participant 2: yeah, the pharmacy

Participant 3: for so many years, because I remember when I was very little I was getting sick on and often and when I grew up after SSS it got serious anytime we go the hospital, it either malaria or fever or something. So mine it took long before

Participant 4: when I went to the first facility I was admitted there I think a week after that I was still not better so I will I was sent here. So like two weeks. Before that it was about three months.

Participant 5: mine it was almost 10 years before I was at the hospital. I was suffering the joint pain was there for like 10 years before I was diagnosed

Participant 6: it took about a week after the symptoms started before I went to nima clinic

Participant 9: When it started that same day I went to the drugstore then they gave me some ointment to apply. It was not working it was Sunday so the Monday or Tuesday I went to the cathedral and They said I should do a lab.so I did it and they transferred me here on Monday. i came but they said they don't work on Monday so I should come on Tuesday.i didn't keep long in the house.

Participant 7: When something is happening to me I don't keep long because I am sickle cell. I rushed here.i was given drugs and told to go and come go and come plus test then they saw it.so me I didn't delay.

Participant 8: When it happened they saw I was pregnant.people always say it's because of the pregnancy and that after three months it will go but it was not going so after I gave birth for about 3 to 4 months was when I went to the hospital so that's been one and half years now.

Participant 10: me I did instantly after I did that test and they saw the rheumatoid factor, I was immediately referred here, rheumatology. Me I come here monthly [redacted] as it happened then he referred me here and I was giving a date.

Interviewer: why did you visit the first place you went to? You said it was a hospital why did you go there?

Participant 1: because I want to know what is wrong with me.

Interviewer: please the pharmacy. Why did you go to the pharmacy?

Participant 2: because I was in pain

Participant 4: I was in pain, very severe pain. My joint was hurting, I was losing hair. I just felt sick so I just had to go and get a solution

Participant 5: I was losing my hair, joint pain, sometimes someone has to bath me, feed me so I had to go the hospital

Participant 6: I became big, and after walking for a while my heart starts to beat really fast

Interviewer: did you visit other facilities?

Participant 1: yes, it is the hospital, one herbal

Interviewer: herbal, traditional, church

Participant 1: all was there

Participant 2: church, pharmacy, hospital

Participant 3: hospital and church

Interviewer: after you stayed home for so long many years, the first facility you said it was the hospital, why did you go to that particular hospital? Which hospital did you go to?

Participant 3: I went there to seek treatment

Participant 1: and finally hospital

Participant 4: NO

Participant 5: AMEN scientific and other herbal hospitals, two herbal hospitals

Participant 6: no

Participant 10: I didn't go anywhere just straight here.

Question 3

Interviewer: what do you understand now about your condition?

Participant 1: it is a chronic disease, manageable but praying for a cure

Participant 2: I know it is manageable

Participant 3: I know it is a chronic disease that can be managed

Participant 4: SLE it has to do with pain like inflammation, the joint or kidneys or any other places

Participant 5: doctor said it is not curable but can be managed with medications

Participant 6: what I know is when you take the medication It suppresses it

Participant 7: I don't know if it is from the family because my mother and father don't have so I don't know.as for my mother she has asthma but my father has nothing so me I don't know.

Participant 8: me I haven't taken it any how cause human beings so far as you are not dead and alive God creates us so far as you are not dead,he hasn't finished creating you.daily, God rubs his fingers around you so until you put your hands on your chest, he has finished creating you.so how he turns turns us is up to him. that is how I have taken it.

Participant 9: me I know it is about our immune system. what I heard is that it is fighting against itself that is what the doctor at nufu clinic said.me I didn't believe because i asked myself that if God created them for me to fight against infections then why is it fighting against itself. i

took it personal because it worried me but when I came here and they explained,I got the understanding.

Participant 10: As I said earlier, aging is part.as human beings grow, your bones become stiff,it is not like when you were a child that your bones is flexible. The more you grow the more your bones become hard.it is something bound to happen so you take it like that and accept it. You only have to counteract, it something like exercise, may be massaging and things just to move account.

Interviewer: where did you receive the information from?

Participant 1: Korle Bu; nurses and doctors

Participant 2: Korle Bu; nurses and doctors

Participant 3: Korle Bu and after that i read more

Participant 4: I go on the internet and check about it

Participant 5: the internet when you check on it the information is very scary but the doctor it is different. So now I have stopped checking. When I was diagnosed I check a lot and I was afraid

Participant 6: here in Korle bu

Participant 7, 8 and 9: one of the nurses.

Participant 10: ok I read about one or two pamphlets which Advice someone how to go about it sometimes too when we come, they tell us one or two in the morning before we see the doctors.

Interviewer: research?

Participant 1 and 2: we also read more

Interviewer: what do you think is causing your condition? Is it let's say, lifestyle, working, ageing, spiritual, is it a curse?

Participant 1:i don't know but after reading about it maybe some drugs or i don't know whether it is hereditary because i don't know any of my family member who is having it so I can't tell about that one. But with medication maybe or stress from work.

Participant 2: me I thought it was my immune system and i also thought it was hereditary

Interviewer: what do you believe is causing it now?

Participant 2: I don't have, like I don't have, I complete, it's in bits. I thought it was spiritual because it very strange it is still there is my mind. I will not lie to you but i also think it is my immune system. That is why I said bit of everything i can't leave the spiritual part out

Participant 3: now I believe it is because my immune system is sometimes fighting my body that's the only reason I have

Participant 10: Exercise is part. Frankly am not the type that's exercises. I have sat for long. human beings if you are there and you are active, most of these things won't attack you and even if it does, it won't be serious like that and our diet play a role.me I strongly believe that our diet plays a role. oh yes and aging .Human beings as I said when you are a child, you are flexible, as you are growing, your waist can't at all.As I said

Participant 8: our diet

Participant 7: As daddy said about the exercise, me I will agree because human beings, if you wake up in the morning and you don't come out of your room and you sit at one place, you get fed up. But if you are able to move and find water to bath and go and come, you exercise, you become light weight. You understand? First, when the thing first happened. I felt it. First I used my two hands but now one of my hands can't go up. You understand? So right now I have become a left handed person. It's my left hand that has strength. So when it happens I came to sickle cell to report. Three days today, they referred me to orthopedic so three days today, came to the clinic and the doctor attended to me. He has given me some medication that I should wait and observe if something happens, I should come if nothing happens and the date is due, I should come so that is what happened to me. Exercises is also part. I don't exercise exercise but when I wake up and walk around, I feel light.

Participant 9: exercise naa

Interviewer: so from what you have told me you all visited other facilities before coming here? Can you walk me through the facilities you have visited?

Participant 1: hospital, herbal, hospital, hospital, hospital, hospital, church, then herbal again, traditional, I went the last one, prayer camp then hospital

Interviewer: Korle Bu?

Participant 1: another hospital then that hospital transferred me to korle bu

Participant 2: pharmacy, hospital, fellowship, hospital

Participant 3: hospital throughout for some years then i was blending the hospital and church. I was at the church and then when it was time for review I will visit the hospital until that hospital referred me to korle bu

Interviewer: what was your experience with the other facilities?

Participant 4: they took care of me very well but they thought it was malaria so they were treating me for malaria

Participant 5: for [redacted] I have been going there for a while they thought it was malaria so they were treating me for malaria. So one day a met a certain doctor and she asked me to stop all the medicine I have been

taking and she asked me to run some test it was very expensive. Then I was diagnosed then I was referred

Participant 6: when I went there they said my blood level is low so they prescribed a medication for me to buy but it did not work so they transferred me

Interviewer: after diagnosis you here have you felt the need to go to other facilities for further treatment?

Participant 1, 2 and 3: no

Participant 4: NO

Participant 5: no, ever since I was diagnosed I have not been to any place again. Its only here

Participant 6: any time I go to another clinic they always say they do not know which drug to give me so now I do not worry myself

Participant 9: Me like this, I have been to a private hospital at [redacted]. So when I came here, I kept long so he will take me there. When I went, they said they don't have a doctor so their name is in Korle-Bu ,so the doctors are at Korle-bu so I have been at Korle-Bu. Private, they can't help me .

Participant 4: Me from korlebu. [redacted]

Participant 5: korlebu here

Participant 4: Even if you take it to another place,I tell them to write on my card for so when I go and see the doctor they are like what are you doing here.this is not your clinic. So I know if I go somewhere, they will sack me so it's korlebu that I'm standing, korlebu here naa.

Participant 10: Korlebu here naa comes in mind.

Participant 9: Me sometimes herbal comes in mind,I want to try but I haven't tried it

Participant 8, 9 and 10: Here's our last stop

Interviewer: why?

Participant 1: because I am strong

Participant 2: because I know that they have the proper facilities and everything to treatment. So I don't think I should go anywhere

Participant 3: no because I think I am getting better treatment here.

Participant 4: here you just come here you get the drug that you are supposed to take. I have gotten sick or any other thing

Participant 5: even if you go there you will still come here

Interviewer: so how do you compare you current treatment to the previous ones, do you think it better here?

Participant 1: far far better

Participant 2: it is better

Participant 3: it is very god here, very very good

Participant 4: this place is better, at least they know what is wrong with you. The other place they don't know what is wrong with you. So this place is better as compared to the other place.

Participant 5: this place is better at least the doctor and nurses they know your condition so they are able to treat you well

Participant 6: this place is better for me. Here the know my condition

Participant 8: Me it's ok for me.

Participant 9: It's okay for me

Participant 10: It is ok for me because my here was steam but now I'm normal. I will come and I will be okay. I feel okay. Yes

Participant 7: Because I couldn't walk. The pains I felt wasn't easy. I used to suffer a lot so when I come here, although I feel some of the pains but am ok.

Interviewer: how do you feel about the outcome?

Participant 1: I don’t know how to grade you, more than good

Participant 2: I am happy within myself

Participant 3: I feel strong with the outcome so I am okay with it

Participant 5: Still I am not feeling well because sometimes the medicine you take, sometime I put it in my palm then I am looking at it, but later on before I will go and it is very stressful, taking medicine everyday it is not easy but still I am taking it hoping one day one day God will heal us from it.

Participant 6: it good for me and when I take the medicine I become strong and I am able to do what I want to do so it good for me. I am looking to God now.

Participant 4: I feel good, I feel better now

Interviewer: do you always take your medications as prescribed by the specialist?

Participant 1: yes but sometimes I don’t when I am travelling

Interviewer: why, why don’t you?

Participant 1: I don’t want to “wewe” on the way

Participant 2: I do but sometimes when there is a big occasion(I do events) sometimes I don’t take it because of two reasons: one

because outside the facilities are not clean I have to go and " wewe" all the time and two I get tired so I have to rest for some few minutes then I will be okay

Participant 3: I take it regularly always, I never forget but sometimes I delay in taking it.

Participant 5: yes

Interviewer: why?

Participant 5: because when i take it I'm relieved from the pain and i am able to do what i am supposed to do

Participant 6: yes, because when I take it I will get the strength to be able to work

Participant 4: yes, because I don't want it to come back. I have to take my medication so that everything will be the same

Participant 7,8,9 and 10 yes

Interviewer: so apart from the prescribed medication from here, do you have other things that you do to help, do you have other medications, do you pray, food supplements, massage?

Participant 1: I take paracetamol

Participant 2: I exercise, and I think exercise and prayer.

Participant 3: I don't have anything I do apart from the medication

Participant 4: I pray every day but I pray everyday but no so seriously like I do not rely on other things

Participant 5: with prayers we pray already so we pray to God so HE delivers us from the condition

Interviewer: what about self-medication?

Participant 5: no, the current medications are already plenty.

Participant 6: no, I do not take other medications. But I pray to God to heal me through my medication

Participant 4: but I hear exercise helps so I exercise too (easy exercise)

Interviewer: Apart from the drugs given to you at the hospital, do you take any other drugs?

Participant 7, 8 and 9: no

Participant 10: Me I'm hypertensive so I take that drugs too

Interviewer: was it also prescribed by a doctor.

Participant 10: Yes. That was the medicine prescribed for me from the previous hospital so anytime I go to the hospital, I make aware that I am hypertensive.

Participant 9: as for para yes

Interviewer: who have you told about your condition, family member, and work colleagues?

Participant 1: everyone around me

Participant 2: not everybody, few family, few friends, everybody does not need to know my condition.

Participant 3: only my family.

Participant 4: my best friend, my parents, my aunties and uncles

Participant 5: only my close family knows, i don't have friends

Participant 6: i have told all my family and friends. They act normal

Participant 10: Someone close to me that knows so those close to me as for them, they know it's for bones sickness that is attacking me.

Participant 9: Apart from my husband I have not told anyone.

Interviewer: Not even a friend or or close family?

Participant 9: No

Participant 10: My wife and my close friends are aware .yeah.

Interviewer: how do they treat you knowing you condition, those who know about it how do they treat you?

Participant 1: they don't treat me bad and creating awareness that is why i am letting them know but they don't treat me bad. But me if you bad i don't care

Participant 2: when you say that they feel sorry for you then you say "oh you don't have to feel sorry for me, I am fine" after I say that then they say ok, they kind of feel sorry for me, when they happen to see, hear or see the symptoms and all that.

Participant 3: they don't treat me any different but sometimes, my mother gets so emotional about the thing and her behavior sometimes i don't like it. I even sometimes regret letting her know this is my illness.

Participant 4: very normal

Participant 5: they act normal

Participant 10: They make us feel comfort. When you are doing something, they know your situation so if you doing something and it's wrong they understand. If your hands is hard or you can't do it, they know that it's because of the sickness so it shouldn't disturb you and

they comfort you that it is because of this and that so have patience,don't do this, do that. Yeah

Participant 8: He knows I can't wash and sometimes cooking and stuff so we are there, we help each other.

Participant 9: Me, the family I'm from, they treat me well. They boil water and send it to the bathroom for me. After bathing I have someone that takes my things, someone that wash my things. I don't go to work, oh I'm in the room. If I say I won't come out, I won't come out. Any type of food will be brought to me so I don't have a problem.

Interviewer: how do others treat, those who do not know about you when they see some physical symptoms, deformity?

Participant 1: I don't care about what they think.

Interviewer: but how do they treat you, do you realize people treating you differently?

Participant 1: I don't know, I don't look at them so I don't know how they are treating me in their head.

Participant 2: for me yes, my cheeks got swollen, it gets to me because it was not how my face was and when they happen to see me the first thing they say is "ooh you have grown big oo, your cheeks have become big, and I'm like because I don't want to tell them what I am going

through, I tell them it is god. And that it is evidence of good living. Meanwhile I know that I am suffering. They treat me like, I am very happy, because I look good and they don't know.

Interviewer: so they didn't see any deformities?

Participant 2: my own was hidden, so they don't read negatively into it. It was a complement

Participant 3: the only thing is that, mine in the morning I become very heavy like I am fat and in the evening you can see that I have come down, slim. So my seamstress likes to make fun of me. She says anytime you come to sow a dress, you come with a different size and when you are coming to try it on, you come with a different size. But she doesn't know and others who also don't know I don't know what they think, but I think they are like "you are always sick" because I have been getting sick one or two times. At church I am very active, but when it becomes very serious, I go for work throughout the week but Sundays I like to rest. The church people will know, why is she not coming to church perhaps she is sick.

Interviewer: so how has your condition affected your ability to do things, physically, emotionally or mentally?

Participant 1: not at all

Participant 2: a bit

Interviewer: physical or emotional?

Participant 2: kind of emotional, because there are periods where I get very emotional and sometimes I don't know you just get very emotional. It is like the world is coming to an end. But you start getting guilty within yourself. But after sometime you are ok. It goes on and off.

Participant 3: it affects me emotionally, sometimes I think about it where could this come from, what are people saying about me when they see me, yes I think about it.

Participant 1: and even child bearing is a problem I think about it small small

Participant 4: when i was sick i wasn't able to go to school, i was always at home feeling weak. I wasn't able to write exam. It has affected me

Interviewer: socially, friends?

Participant 4: i wasn't able to go out also. I was always inside

Interviewer: how did it make you feel?

Participant 4: i felt really bad. Not being able to go out to chill

Participant 5: with this condition if you should think about it, hmmm. So some of the thing you ignore and move on

Interviewer: what things?

Participant 5: like sometimes when want to do something, even when you work and you feel tired then it seems like you are feeling the sickness again then you are in pain. So have to relax.

Interviewer: how do you feel?

Participant 5: i just sleep. I don’t go out

Interviewer: socially, friends?

Participant 5: i don’t have friends. I normally like staying at home. During my school time i will stay at home for sometimes one month and not go to school

Interviewer: relationship, has it affected your relationship?

Participant 4: i have not been in a relationship for a some time now

Participant 5: very, knowing your condition, the money you spend definitly the person will go. But we are still hoping

Interviewer: has it happened?

Participant 5: yes, twice

Interviewer: how has your condition affected you?

Participant 6: it hasn’t affected me

Interviewer: are you able to everything the way you want

Participant 6: yes

Interviewer: are you married

Participant 6: no.

Interviewer: are you dating?

Participant 6: yes

Interviewer: does he know your condition?

Participant 6: yes

Interviewer: how does he treat you?

Participant 6: normal, i want to quite because i of my condition. He does not treat me the way he is suppose to treat me. When he is suppose to help me he doesn't. When i ask for something he gets angry. And we have 3 children

Interviewer: are u afraid when it comes to child bearing?

Participant 5: yes, i am 32. And i have given birth, that will push men away maybe they will want a child. When it comes to money someone can help

Participant 4: yes, i want to give birth, just one. I heard some people find it difficult to give birth.

Interviewer: do you believe you will find someone who will accept you?

Participant 4: i hope so

Participant 5: i have forgotten about that.

Interviewer: so how do you cope with it, be it physical or emotional?

Participant 1: As far as there is life there is everything, so that is what is keeping me going.

Participant 2: first of all god, because I take god very seriously and two my family they have been so supportive I couldn't have done it without them also.

Participant 3: the hope of life, once I am alive there is still hope.

Participant 5: it hasn't been long since this happened but it's like god has given me the courage, before i think a lot about things like this and i am unable to do what i am suppose to do. But this(relationship) i don't this about it, if he is someone i am meant to be with he will stay. Not everyone is meant to marry or give birth

Participant 4: just live each day hoping and praying that everthing goes well

Interviewer: how do you perceive the future?

Participant 4: i just pray everyday that it does not reoccur and come severely like it used to. I just hope the future will be bright.

Participant 5: since I have god I have a future

Participant 6: I don’t have a problem. It is normal. When I see my children I become happy

Participant 7: I look up to GOD

Participant 8: I cope well because when I didn’t have it I was sickle cell. I’m strong. The only problem is when the crisis come, I go on medication I live alright. I work at first they knew I was smart and brave and now when I can’t do something if you compare , they say I have done it before so they don’t worry me so I live on my own. The work I do what I can do and stop the one I can’t. When the white person sees me they don’t talk.

Participant 9: Sometimes I feel bad because me I don’t want anybody to do something for me and even if the person does the thing for me, how you want it the person won’t do it like that so it makes me feel bad sometimes I ask why did I pass through this. Sometimes

Participant 10: Now that they have diagnosed and know that it’s rheumatoid arthritis, in fact when it started I was seeing symptoms of stroke and others but when I came here and they diagnosed means I did the test and I saw that wasn’t what was going on , so I know how to cope that this and that is what is going on with me.
