# Supplementary material for: Health-seeking behaviour, referral patterns and associated factors among patients with autoimmune rheumatic diseases in Ghana: A cross-sectional mixed method study
Source: PLoS One. 2022 Sep 12;17(9):e0271892. doi: 10.1371/journal.pone.0271892 (PMC9467363; doi:10.1371/journal.pone.0271892)
Supplement: S5 Appendix — (ZIP) [file pone.0271892.s009.zip › AUDIO TRANSCRIPTION 23.pdf]

AUDIO TRANSCRIPTION NO. 23

INTERVIEWER: First question, what do you do when you are usually not well?

PARTICIPANT 1: Go and see the doctor.

PARTICIPANT 2: me sometimes I go for medications before I go and see the doctor. I go to the pharmacy

PARTICIPANT 3: I take some medications

INTERVIEWER: do you go to the pharmacy or you buy it on your own?

PARTICIPANT 3: no, I take the ones I was given at the hospital

INTERVIEWER: but normally what do you do?

PARTICIPANT 3: I don't go anywhere, I just get some medication at the pharmacy

INTERVIEWER: Please why do you take that action? Why do you go to the hospital?

PARTICIPANT 1: to get better

INTERVIEWER: What informs that decision?

PARTICIPANT 1: seeing the doctor I think is the best,

INTERVIEWER: Please why do you go and see the pharmacist?

PARTICIPANT 2: I go so that they give me some medications then I may be relieved, because when you feel the pains you have to be relieved from it

PARTICIPANT 3: because of the pains I feel, I want it to reduce. That is why I came to the clinic

INTERVIEWER: who determines where you go?

PARTICIPANT 1: Like relatives or?

INTERVIEWER: Yes yourself, relatives or friend?

PARTICIPANT 1: Yes my dad

INTERVIEWER: Why does he help inform your decisions?

PARTICIPANT 1: he is the head, normally if you are not well he will say you should go to the hospital

PARTICIPANT 2: my mum

INTERVIEWER: Why does she?

PARTICIPANT 2: sometimes I will go with her, other times she will go and get it for me

PARTICIPANT 3: no one, I go on my own

INTERVIEWER: about your current condition, please what is your current diagnosis?

PARTICIPANT 1: SLE

INTERVIEWER: have you heard about it before?

PATICIPANT 1: it was once I was watching a program and the person had it and she was losing her hair. And that was the first time I heard about it

INTERVIEWER: what program was it?

PARTICIPANT 1: America's next top model

INTERVIEWER: what information did you get?

PARTICIPANT1: it was like the condition had to with, she was having joint pains also and she lost most of her because of that so that is what I got to know about the condition

INTERVIEWER: Madam, please have you heard about your condition? What is your diagnosis?

PARTICIPANT 2: SLE

INTERVIEWER: have you heard about it before?

PARTICIPANT 2: NO IT WAS WHEN I WAS DIAGNOSED THAT I CAME TO KNOW ABOUT IT

INTERVIEWER: what did you know about your condition before you were diagnosed?

PARTICIPANT 3: SLE, NO I don't know anything about it

INTERVIEWER: WHAT DO YOU THINK caused the problem?

PARTICIPANT 1: I don't know

INTERVIEWER: DO YOU HAVE ANY BELIEVES ABOU IT, any ideas? Madam what do you think caused it?

PARTICIPANT 2: THE doctors said they don't know so me myself I don't know

INTERVIEWER: Maybe your lifestyle,

PARTICIPANT 2: sometimes they said it is your environment in which you live, sometimes depression but the actual cause they don't know

PARTICIPANT 3: I Heard its like someone, I don't know how to describe it, it happens to someone who does not rest and does everything fast fast

INTERVIEWER: what about spiritual cause?

PARTICIPANT 3: no

PARTICIPANT 2: first the way the sickness is you will think maybe someone is causing it. You will be going to all sort of places but then you will have to come to the hospital

PARTICIPANT 1: no

INTERVIEWER: PLEASE WHERE DID YOU GO WHEN THE SYMPTOMS STARTED?

PARTICIPANT 1: TO THE hospital

INTERVIEWER: WHICH HOSPITAL?

PARTICIPANT 1 : TWO HOSPITALS

PARTICIPANT 2:A LOT

INTERVIEWER: CAN YOU MENTION SOME?

PARTICIPANT 2: THE ONE THAT I WAS REFERRED FROM WAS 37

INTERVIEWER: BEFORE 37 WHERE DID YOU GO?

PARTICIPANT 2: [redacted], [redacted], I can't mention all they are many, more than 10. Even herbal hospitals, plenty. It was like malaria, when you go they give you the medicine then it will come back again so I stopped going to the hospital. So the medicine that I know if I take I will be ok then I will go to the pharmacy but later on I said no I have to go to the hospital. That is why I went to [redacted] then I was diagnosed with SLE then I came here

INTERVIEWER: PLEASE WHERE DID YOU GO WHEN THE SYMPTOMS STARTED?

PARTICIPANT 3: I went to around about, [redacted], afterward I came to [redacted] clinic then they transferred me here

INTERVIEWER: DID YOU VISIT A HERBAL HOSPITAL?

PARTICIPANT ALL: NO

INTERVIEWER: HOW LONG DID IT TAKE YOU TO VISIT THESE FACILITIES, BETWEEN THE ONSET AND SYPMTOMS ?

PARTICIPANT 1: when I went to the first facility I was admitted there I think a week after that I was still not better so I will I was sent here. So like two weeks. Before that it was about three months.

PARTICIPANT 2: mine it was almost 10 years before I was at the hospital. I was suffering the joint pain was there for like 10 years before I was diagnosed

PARTICIPANT 3: it took about a week after the symptoms started before I went to [redacted] clinic

INTERVIEWER: What prompted you to go there?

PARTICIPANT 1: I was in pain, very severe pain. My joint was hurting, I was losing hair. I just felt sick so I just had to go and get a solution

PARTICIPANT 2: I was losing my hair, joint pain, sometimes someone has to bath me, feed me so I had to go the hospital

PARTICIPANT 3: I became big, and after walking for a while my heart starts to beat really fast

INTERVIEWER: PLEASE DID YOU VISIT OTHER FACILITIES ?

PARTICIPANT 1: NO

PARTICIPANT 2: AMEN scientific and other herbal hospitals, two herbal hospitals

PARTICIPANT 3: no

INTERVIEWER: so now that you have been diagnosed, do have and idea what the condition is about?

PARTICIPANT 1: SLE it has to do with pain like inflammation, the joint or kidneys or any other places

PARTICIPANT 2: doctor said it is not curable but can be managed with medications

PARTICIPANT 3: what I know is when you take the medication It suppresses it

INTERVIEWER: where did you receive much of the information?

PARTICIPANT 1: I go on the internet and check about it

PARTICIPANT 2: the internet when you check on it the information is very scary but the doctor it is different. So now I have stopped checking. When I was diagnosed I check a lot and I was afraid

PARTICIPANT 3: here in Korle bu

INTERVIEWER: WHAT WAS YOUR EXPERIENCE WITH THE OTHER FACILITIES?

PARTICIPANT 1: they took care of me very well but they thought it was malaria so they were treating me for malaria

PARTICIPANT 2: for [redacted] I have been going there for a while they thought it was malaria so they were treating me for malaria. So one day a met a certain doctor and she asked me to stop all the medicine I have been taking and she asked me to run some test it was very expensive. Then I was diagnosed then I was referred

PARTICIPANT 3: when I went there they said my blood level is low so they prescribed a medication for me to buy but it did not work so they transferred me

INTERVIEWER: After you were diagnosed and you have been coming here have you felt the need to go to other facilities?

PARTICIPANT 1: NO

INTERVIEWER: WHY?

PARTICIPANT 1: here you just come here you get the drug that you are supposed to take. I have gotten sick or any other thing

INTERVIEWER: What about traditional, herbal?

PARTICIPANT 1: no

PARTICIPANT 2:no, ever since I was diagnosed I have not been to any place again. Its only here

INTERVIEWER: WHY?

PARTICIPANT 2: even if you go there you will still come here

PARTICIPANT 3: any time I go to another clinic they always say they do not know which drug to give me so now I do not worry myself

INTERVIEWER: SO HOW DO YOU COMPARE YOUR TREATMENT HERE WITH OTHERS?

PARTICIPANT 1: this place is better, at least they know what is wrong with you. The other place they don't know what is wrong with you. So this place is better as compared to the other place.

PARTICIPANT 2: this place is better at least the doctor and nurses they know your condition so they are able to treat you well

PARTICIPANT 3: this place is better for me. Here the know my condition

INTERVIEWER : SO HOW DO YOU FEEL ABOUT THE FACT THAT YOU ARE HERE AND YOU ARE GIVEN THE TREATMENT ND YOU ARE MANAGING?

PARTICIPANT 2: Still I am not feeling well because sometimes the medicine you take, sometime I put it in my palm then I am looking at it, but later on before I will go and it is very stressful, taking medicine everyday it is not easy but still I am taking it hoping one day one day God will heal us from it.

PARTICIPANT 3: it good for me and when I take the medicine I become strong and I am able to do what I want to do so it good for me. I am looking to God now.

PARTICIPANT 1: I feel good, I feel better now

INTERVIEWER: PLEASE DO YOU ALWAYS TAKE YOUR MEDICATION AS PRESCRIBED?

PARTICIPANT 2: yes

INTERVIEWER: WHY?

PARTICIPANT 2:BECAUSE WHEN I TAKE IT IM RELIEVED FROM THE PAIN AND I AM ABLE TO DO WHAT I AM SUPPOSE TO DO

PARTICIPANT 3: YES, BECAUSE when I take it I will get the strength to be able to work

PARTICIPANT 1 yes, because I don't want it to come back. I have to take my medication so that everything will be the same

INTERVIEWER: apart from the prescribed medication do you use other self-help practices, maybe self-medication, prayer?

PARTICIPANT 1: I pray every day but I pray everyday but no so seriously like I do not rely on other things

PARTICIPANT 2: with prayers we pray already so we pray to God so HE delivers us from the condition

INTERVIEWER: WHAT ABOUT SELF-MEDICATION?

PARTICIPANT 2 : NO, the current medications are already plenty.

PARTICIPANT 3: no, I do not take other medications. But I pray to God to heal me through my medication

PARTICIPANT 1: but I hear exercise helps so I exercise too (easy exercise)

INTERVIEWER: WHO HAVE YOU TOLD ABOUT YOUR ILLNESS?

PARTICIPANT 1: MY best friend, my parents, my aunties and uncles

INTERVIEWER: HOW DO THEY RELATE WITH YOU AFTER FINDING OUT?

PARTICIPANT 1: VERY NORMAL

PARTICIPANT 2: ONLY MY CLOSE FAMILY KNOWS, I DON'T HAVE FRIENDS

INTERVIEWER: HOW DO THEY RELATE WITH YOU AFTER FINDING OUT?

PARTICIPANT 2: THEY ACT NORMAL

PARTICIPANT 3: I HAVE TOLD ALL MY FAMILY AND FRIENDS. THEY ACT NORMAL

INTERVIEWER: HOW DO THEY RELATE WITH YOU AFTER FINDING OUT?

PARTICIPANT 3THEY ACT NORMAL

INTERVIEWER: HOW HAS YOUR CONDITION AFFECTED YOUR ABILITY TO DO THINGS?

PARTICIPANT 1: WHEN I WAS SICK I WASN'T ABLE TO GO TO SCHOOL, I WAS ALWAYS AT HOME FEELING WEAK. I WASN'T ABLE TO WRITE EXAM. IT HAS AFFECTED ME

INTERVIEWER: SOCIALLY, FRIENDS?

PARTICIPANT 1: I WASN'T ABLE TO GO OUT ALSO. I WAS ALWAYS INSIDE

INTERVIEWER: HOW DID IT MAKE YOU FEEL?

PARTICIPANT 1: I FELT REALLY BAD. NOT BEING ABLE TO GO OUT TO CHILL

INTERVIEWER: HOW HAS YOUR CONDITION AFFECTED YOUR ABILITY TO DO THINGS

PARTICIPANT 2: WITH THIS CONDITION IF YOU SHOULD THINK ABOUT IT, HMMM. SO SOME OF THE THING YOU IGNORE AND MOVE ON

INTERVIEWER: WHAT THINGS?

PARTICIPANT 2: LIKE SOMETIMES WHEN WANT TO DO SOMETHING, EVEN WHEN YOU WORK AND YOU FEEL TIRED THEN IT SEEMS LIKE YOU ARE FEELING THE SICKNESS AGAIN THEN YOU ARE IN PAIN. SO HAVE TO RELAX.

INTERVIEWER: HOW DO YOU FEEL?

PARTICIPANT 2: I JUST SLEEP. I DON'T GO OUT

INTERVIEWER: SOCIALLY, FRIENDS?

PARTICIPANT 2: I DON'T HAVE FRIENDS. I NORMALLY LIKE STAYING AT HOME. DURING MY SCHOOL TIME I WILL STAY AT HOME FOR SOMETIMES ONE MONTH AND NOT GO TO SCHOOL

INTERVIEWER: RELATIONSHIP, HAS IT AFFECTED YOUR RELATIONSHIP?

PARTICIPANT 2: VERY, KNOWING YOUR CONDITION, THE MONEY YOU SPEND DEFINITLY THE PERSON WILL GO. BUT WE ARE STILL HOPING

INTERVIEWER: HAS IT HAPPENED?

PARTICIPANT 2: YES, TWICE

INTERVIEWER: RELATIONSHIP, HAS IT AFFECTED YOUR RELATIONSHIP?

PARTICIPANT 1: I HAVE NOT BEEN IN A RELATIONSHIP FOR A SOME TIME NOW

INTERVIEWER: HOW HAS YOUR CONDITION AFFECTED YOU?

PARTICIPANT 3: IT HASN'T AFFECTED ME

INTERVIEWER: ARE YOU ABLE TO EVERYTHING THE WAY YOU WANT

PARTICIPANT 3: YES

INTERVIEWER: ARE YOU MARRIED

PARTICIPANT 3: NO.

INTERVIEWER: ARE YOU DATING?

PARTICIPANT: 3 YES

INTERVIEWER: DOES HE KNOW YOUR CONDITION?

PARTICIPANT 3: YES

INTERVIEWER: HOW DOES HE TREAT YOU?

PARTICIPANT 3: NORMAL, I WANT TO QUIT BECAUSE I OF MY CONDITION. HE DOES NOT TREAT ME THE WAY HE IS SUPPOSE TO TREAT ME. WHEN HE IS SUPPOSE TO HELP ME HE DOESN'T. WHEN I ASK FOR SOMETHING HE GETS ANGRY. AND WE HAVE 3 CHILDREN

INTERVIEWER: ARE U AFRAID WHEN IT COMES TO CHILD BEARING?

PARTICIPANT 2: YES, I AM 32. AND I HAVE GIVEN BIRTH, THAT WILL PUSH MEN AWAY MAYBE THEY WILL WANT A CHILD. WHEN IT COMES TO MONEY SOMEONE CAN HELP

PARTICIPANT 1: YES, I WANT TO GIVE BIRTH, JUST ONE. I HEARD SOME PEOPLE FIND IT DIFFICULT TO GIVE BIRTH.

INTERVIEWER: DO YOU BELIEVE YOU WILL FIND SOMEONE WHO WILL ACCEPT YOU?

PARTICIPANT 1: I HOPE SO

PARTICIPANT 2: I HAVE FORGOTTEN ABOUT THAT.

INTERVIEWER: HOW DO YOU COPE?

PARTICIPANT 2: IT HASN'T BEEN LONG SINCE THIS HAPPENED BUT IT'S LIKE GOD HAS GIVEN ME THE COURAGE, BEFORE I THINK A LOT ABOUT THINGS LIKE THIS AND I AM UNABLE TO DO WHAT I AM SUPPOSE TO DO. BUT THIS(RELATIONSHIP) I DON'T THIS ABOUT IT, IF HE IS SOMEONE I AM MEANT TO BE WITH HE WILL STAY. NOT EVERYONE IS MEANT TO MARRY OR GIVE BIRTH

INTERVIEWER: HOW DO YOU PERCEIVE THE FUTURE?

PARTICIPANT 2: SINCE I HAVE GOD I HAVE A FUTURE

INTERVIEWER: HOW DO YOU COPE?

PARTICIPANT 1: JUST LIVE EACH DAY HOPING AND PRAYING THAT EVERTHING GOES WELL

INTERVIEWER: HOW DO YOU PERCEIVE THE FUTURE?

PARTICIPANT 1: I JUST PRAY EVERYDAY THAT IT DOES NOT REOCCUR AND COME SEVERLLY LIKE IT USE TO. I JUST HOPE THE FUTURE WILL BE BRIGHT.

INTERVIEWER: HOW DO YOU PERCEIVE THE FUTURE?

PARTICIPANT: I DON'T HAVE A PROBLEM. IT IS NORMAL. WHEN I SEE MY CHILDREN I BECOME HAPPY
