# Supplementary material for: Health-seeking behaviour, referral patterns and associated factors among patients with autoimmune rheumatic diseases in Ghana: A cross-sectional mixed method study
Source: PLoS One. 2022 Sep 12;17(9):e0271892. doi: 10.1371/journal.pone.0271892 (PMC9467363; doi:10.1371/journal.pone.0271892)
Supplement: S5 Appendix — (ZIP) [file pone.0271892.s009.zip › AUDIO TRANSCRIPTION 27.pdf]

AUDIO TRANSCRIPTION 27

QUESTION 1

Interviewer: what do you do when you are usually not well?

Participant 1: I sleep

Participant 2: I rest and also take medication because i am too quick to take a medication like pain killer

Participant 3: I rest but sometimes I have to go on with my normal duties. Sometimes I try hard even if I am sick I go on with my normal duties.

Interviewer: so why do you take the action you take, why do you sleep?

Participant 1: so that I will not stress myself

Participant 2: I don't want the pain to increase more that's why i take the medication.

Participant 3: with me I have to work for myself and cater for myself, so nothing can keep me down even sickness cannot keep me down

Interviewer: so you for instance, you sleeping you decide on your own to sleep or do other people advise you to sleep?

Participant 1: on my own

Participant 2: for me, on my own

Participant 3: yes that is my own decision

QUESTION 2

Interviewer: about your current illness have you heard about it before you were diagnosed?

Participant 1: no

Interviewer: nothing at all? No form knowledge about it?

Participant 1: no

Participant 2: I have heard about it, but i didn’t have so much knowledge about it

Interviewer: what did you hear about it?

Participant 2: what I heard is that it is not curable, it can be managed but i didn’t know, I didn’t know, how will I explain it, I didn’t know too much

Interviewer: where did you hear about it?

Participant 2: on tv

Participant 3: I didn’t know anything about it

Interviewer: what do you think caused your problem?

Participant 1: I don’t know what caused it

Interviewer: you don’t believe it is ageing or work or stress, lifestyle nothing?

Participant 1: no

Participant 2: me I thought two things; one, I thought it was hereditary and i thought it was my immune system being damaged

Interviewer: before you were diagnosed?

Participant 2: yes before

Participant 3: I didn’t know what the cause was until recently that I read about it, some possible things could be the cause

Interviewer: where did you go to when the symptoms started the first time?

Participant all: hospital

Interviewer: how long did it take you to go to this facility you mentioned?

Participant 1: I don't remember

Interviewer: can you tell me in months or years?

Participant 1: when it started I was going frequently sometimes the symptoms comes and goes

Interviewer: so when it started it didn't take so long

Participant 1: no

Participant 2: for me six months but before that six months i was taking pain killers

Interviewer: so you will say the first place you went to was the pharmacy?

Participant 2: yeah, the pharmacy

Participant 3: for so many years, because I remember when I was very little I was getting sick on and often and when I grew up after SSS it got serious anytime we go the hospital, it either malaria or fever or something. So mine it took long before

Interviewer: why did you visit the first place you went to? You said it was a hospital why did you go there?

Participant 1: because I want to know what is wrong with me.

Interviewer: please the pharmacy. Why did you go to the pharmacy?

Participant 2: because I was in pain

Interviewer: after you stayed home for so long many years, the first facility you said it was the hospital, why did you go to that particular hospital? Which hospital did you go to?

Participant 3: I went there to seek treatment

Interviewer: did you visit other facilities?

Participant 1: yes, it is the hospital, one herbal

Interviewer: herbal, traditional, church

Participant 1: all was there

Participant 2: church, pharmacy, hospital

Participant 3: hospital and church

Participant 1: and finally hospital

Participant 2: yes

Question 3

Interviewer: what do you understand now about your condition?

Participant 1: it is a chronic disease, manageable but praying for a cure

Participant 2: I know it is manageable

Participant 3: I know it is a chronic disease that can be managed

Interviewer: where did you receive the information from?

Participant 1: Korle Bu; nurses and doctors

Participant 2: Korle Bu; nurses and doctors

Participant 3: Korle Bu and after that I read more

Interviewer: research?

Participant 1 and 2: we also read more

Interviewer: what do you think is causing your condition? Is it let's say, lifestyle, working, ageing

Interviewer: spiritual, is it a curse?

Participant 1:i don't know but after reading about it maybe some drugs or i don't know whether it is hereditary because i don't know any of my family member who is having it so I can't tell about that one. But with medication maybe or stress from work.

Participant 2: me I thought it was my immune system and i also thought it was hereditary

Interviewer: what do you believe is causing it now?

Participant 2: I don't have, like I don't have, I complete, it's in bits. I thought it was spiritual because it very strange it is still there is my mind. I will not lie to you but i also think it is my immune system. That is why I said bit of everything i can't leave the spiritual part out

Participant 3: now I believe it is because my immune system is sometimes fighting my body that's the only reason I have

Interviewer: so from what you have told me you all visited other facilities before coming here? Can you walk me through the facilities you have visited?

Participant 1: hospital, herbal, hospital, hospital, hospital, hospital, church, then herbal again, traditional, I went the last one, prayer camp then hospital

Interviewer: Korle Bu?

Participant 1: another hospital then that hospital transferred me to korle bu

Participant 2: pharmacy, hospital, fellowship, hospital

Participant 3: hospital throughout for some years then i was blending the hospital and church. I was at the church and then when it was time for review i will visit the hospital until that hospital referred me to korle bu

Interviewer: after diagnosis you here have you felt the need to go to other facilities for further treatment?

Participant all: no

Interviewer: why?

Participant 1: because I am strong

Participant 2: because I know that they have the proper facilities and everything to treatment. So I don't think I should go anywhere

Participant 3: no because I think I am getting better treatment here.

Interviewer: so how do you compare you current treatment to the previous ones, do you think it better here?

Participant 1: far far better

Participant 2: it is better

Participant 3: it is very god here, very very good

Interviewer: how do you feel about the outcome?

Participant 1: I don't know how to grade you, more than good

Participant 2: I am happy within myself

Participant 3: I feel strong with the outcome so I am okay with it

Interviewer: do you always take your medications as prescribed by the specialist?

Participant 1: yes but sometimes I don't when I am travelling

Interviewer: why, why don't you?

Participant 1: I don't want to "wewe" on the way

Participant 2: I do but sometimes when there is a big occasion(I do events) sometimes I don't take it because of two reasons: one because outside the facilities are not clean I have to go and " wewe" all the time and two I get tired so I have to rest for some few minutes then I will be okay

Participant 3: I take it regularly always, I never forget but sometimes I delay in taking it.

Interviewer: so apart from the prescribed medication from here, do you have other things that you do to help, do you have other medications, do you pray, food supplements, massage?

Participant 1: I take paracetamol

Participant 2: I exercise, and I think exercise and prayer.

Participant 3: I don't have anything I do apart from the medication

Interviewer: who have you told about your condition, family member, and work colleagues?

Participant 1: everyone around me

Participant 2: not everybody, few family, few friends, everybody does not need to know my condition.

Participant 3: only my family.

Interviewer: how do they treat you knowing you condition, those who know about it how do they treat you?

Participant 1: they don't treat me bad and creating awareness that is why i am letting them know but they don't treat me bad. But me if you bad i don't care

Participant 2: when you say that they feel sorry for you then you say "oh you don't have to feel sorry for me, I am fine"

Interviewer: so do they treat you different?

Participant 2: after I say that then they say ok, they kind of feel sorry for me, when they happen to see, hear or see the symptoms and all that.

Participant 3: they don't treat me any different but sometimes, my mother gets so emotional about the thing and her behavior sometimes i don't like it. I even sometimes regret letting her know this is my illness.

Interviewer: how do others treat, those who do not know about you when they see some physical symptoms, deformity?

Participant 1: I don't care about what they think.

Interviewer: but how do they treat you, do you realize people treating you differently?

Participant 1: I don't know, I don't look at them so I don't know how they are treating me in their head.

Participant 2: for me yes, my cheeks got swollen, it gets to me because it was not how my face was and when they happen to see me the first thing they say is "ooh you have grown big oo, your cheeks have become big, and I'm like because I don't want to tell them what I am going through, I tell them it is god. And that it is evidence of good living. Meanwhile I know that I am suffering. They treat me like, I am very happy, because I look good and they don't know.

Interviewer: so they didn't see any deformities?

Participant 2: my own was hidden, so they don't read negatively into it. It was a complement

Participant 3: the only thing is that, mine in the morning I become very heavy like I am fat and in the evening you can see that I have come down, slim. So my seamstress likes to make fun of me. She says anytime you come to sow a dress, you come with a different size and when you are coming to try it on, you come with a different size. But she doesn't know and others who also don't know I don't know what they think, but I think they are like "you are always sick" because I have been getting sick one or two times. At church I am very active, but when it

becomes very serious, I go for work throughout the week but Sundays I like to rest. The church people will know, why is she not coming to church perhaps she is sick.

Interviewer: so how has your condition affected your ability to do things, physically, emotionally or mentally?

Participant 1: not at all

Participant 2: a bit

Interviewer: physical or emotional?

Participant 2: kind of emotional, because there are periods where I get very emotional and sometimes I don't know you just get very emotional. It is like the world is coming to an end. But you start getting guilty within yourself. But after sometime you are ok. It goes on and off.

Participant 3: it affects me emotionally, sometimes I think about it where could this come from, what are people saying about me when they see me, yes I think about it.

Participant 1: and even child bearing is a problem I think about it small small

Interviewer: so how do you cope with it, be it physical or emotional?

Participant 1: As far as there is life there is everything, so that is what is keeping me going.

Participant 2: first of all god, because I take god very seriously and two my family they have been so supportive I couldn't have done it without them also.

Participant 3: the hope of life, once I am alive there is still hope.
