# Supplementary material for: Health-seeking behaviour, referral patterns and associated factors among patients with autoimmune rheumatic diseases in Ghana: A cross-sectional mixed method study
Source: PLoS One. 2022 Sep 12;17(9):e0271892. doi: 10.1371/journal.pone.0271892 (PMC9467363; doi:10.1371/journal.pone.0271892)
Supplement: S5 Appendix — (ZIP) [file pone.0271892.s009.zip › AUDIO TRANSCRIPTION 25.pdf]

Question 1

Interviewer: What do you do when you are not well?

Participant: I go to the hospital when I am not well

Interviewer: Why did you take this arrangement?

Participant: well, when you are not well, the hospital is the only place the best place to be diagnosed, that is why I came here.

Interviewer: Why did you take that decision?

Who advised you on this decision, did you take a personal decision or it was influenced by someone else?

Participant: I didn't take the decision personally, I was referred to this place. When I went to the hospital I was diagnosed before referred to korle-bu

Question 2

Interviewer: concerning your problem?

2a. Have you ever heard about this problem?

Participant: when I met them, I was asked to go for an x-ray and I went to ECG and the doctor told me that my heart was enlarged so I was to go to cardio. So when I came to cardio I was referred to pneumatology department. So that's why I came here.

Before you realized you had rheumatoid arthritis, did you ever hear anyone make mention of it?

Participant: No I never heard anyone speak about it.

2b. **Interviewer**: What did you know about this condition?

And who informed you about it?

2c. **Interviewer**: Where did you get the explanation of the condition from?

Participant: When I came to cardio, after diagnosis they realized my skin was dry and they didn't understand the reasons for that. So they decided to refer me to the rheumatology department.

Interviewer: What do you think caused you condition?

Participant: I didn't know the exact cause of the condition, so I asked the doctor and he explained that it was the body's reaction. With the first condition, I knew they said my heart was enlarged and when I came here I was told about this condition as well. I find it difficult contracting and relaxing the muscle in the arm.

I don't know the exact cause of this condition.

Interviewer: Do you think it is a spiritual condition?

Participant: No, I never saw this condition as spiritual. Because I didn't think the enlargement of the heart was done by someone. So I never looked at it as spiritual.

Interviewer: Where did you first go before seeing these signs?

Participant: I went to a clinic known as [redacted] in [redacted] I went for a lab test but I realized I was still not fine so I went to [redacted] in [redacted], after the test I realized I was still not fine. So I finally went to a private hospital. I went there this time to check on my ears but the doctor realized that I was not fine so I told him all that I felt. So he asked me to go for an ECG test. After the test, he asked me to go for an x-ray as well. After going through the result he asked me to come to korle-bu.

Interviewer: So you didn't go to any prayer to find out what was happening to you.

Participant: No, we go to church no because of our health issues.

Interviewer: After realizing the condition of your health, how many days did it take you to go the health facility?

Participant: when this condition started, within two weeks I realized I wasn't well. I suspected it to be malaria so I went for a test and I was told It wasn't malaria. So I was advised by a friend to go to a government hospital. So I decided to go to a government hospital, after receiving treatment I realized that I was still not well.

2d. **Interviewer**: What made you go to the hospital?

Participant: I went there purposely because of my ears. I had lots of pains in my ears. A friend recommended a doctor to me at [redacted]. So after the doctor checked my ears, he realized I was not healthy and so he asked me to run some test. After the test he referred me to korle-bu.

Interviewer: Did you ever receive treatment from a herbalist or a pastor?

Participant: No I didn't. I never thought about going for treatment from a herbalist. I didn't specially visit any prayer camp for treatment. I knew my health would be restored when I go to the hospital. The prayer camp is purposely for prayer, but the medicine to restore the health is given to the doctor.

__**Question 3**__

3a.**Interviewer**: what understanding do you have concerning your health after the doctor diagnosed you?

Participant: I see an improvement in my health. I'm looking forward to putting on some weight.

3b.**Interviewer**: Where did you receive most of your explanations concerning your condition from?

3a. **Interviewer:** Did you go elsewhere for treatment before coming here?

And how was the place?

Participant: I realized my condition wasn't getting any better, that's why I came here.

Interviewer: Would you want to go elsewhere to receive treatment?

Participant: No, for now I don't have any health facility in mind. I would want to receive my treatment from here. So I would report when a date is given to me. I see an improvement in my health so I would want to keep receiving treatment from this facility.

Interviewer: what do you mean by improvement?

Participant: The condition caused a lot of unpleasing reactions in my body but now I see a lot of change, I see an improvement.

Interviewer: What is the reason?

Participant: I had a swollen face and leg but now I can see that it's back to its normal state

Interviewer: How do you see how we treat patients here as compared to other health facilities?

Participant: I see the procedure to be normal, you come for your card then the doctor attends to you so I know everything is going on well.

Interviewer: How do you see the effects of the condition?

Participant: I had a swollen face and leg, my mouth decreased in size so i found it difficult putting food into my mouth. I realized that the hair around my ear was like that of an albino. I have realized that it's all gone back to its natural state. I can now open my mouth wide. I also had heartburns when I ate any food, my system couldn't digest the food properly. But now I see a massive improvement.

Interviewer: Do you take your medication as prescribed by the doctor?

Participant: Yes as the doctor prescribed.

Interviewer: Do you take any other drug apart from what is prescribed to you by the doctor?

Participant: No, I am scared of taking in another medicine that is not prescribed by the doctor. I felt uneasy some time ago and I decided to take in paracetamol, after taking in the drug I vomited on countless times so I had fear for taking in paracetamol. The last time I came here and complained of body pains I was given a pain killer and I felt ok after taking it.

Question 4

4a.**Interviewer**: Who else have you discussed your condition with?

Participant: My children are informed about my health condition. My husband is also aware about my condition, he was also not well and I was attending to him when I lost him. After his death, I have been in this condition for two years. I haven't discussed this issue with any of my family members.

Interviewer: How did people relate to you when you got into this condition?

Participant: when I got into this condition I lost a lot of weight so people stared at me when they met me. They could stare at me for a very long period. So I had to stop going to church because how they stared at me made me uncomfortable. I was not frightened about the condition else I would have died.

4b. **Interviewer**: How did people live with you when you found yourself in this condition?

Participant: I live peacefully with everyone around me. Certain times I even take the bathe of my grandchildren. I can even cook at home.

Question 5

5a. **Interviewer**: What are the effects of the condition on your mind, body and interaction with people?

Participant: it has decreased the rate at which I did business and so it has decreased my business. People don't patronize my products as before and so I have to get people to work for me.

Interviewer: Who have you discussed this condition with?

Interviewer: How are you coping with the effects of the condition on your life?

Participant: For now I don't turn my attention to the condition and because of this I am free. I think less about the condition.
