# Supplementary material for: Health-seeking behaviour, referral patterns and associated factors among patients with autoimmune rheumatic diseases in Ghana: A cross-sectional mixed method study
Source: PLoS One. 2022 Sep 12;17(9):e0271892. doi: 10.1371/journal.pone.0271892 (PMC9467363; doi:10.1371/journal.pone.0271892)
Supplement: S5 Appendix — (ZIP) [file pone.0271892.s009.zip › AUDIO 30.pdf]

AUDIO 30

INTERVIEWER – PLEASE WHAT DO YOU DO WHEN YOU ARE USUALLY NOT WELL

FEMALE INTERVIEWEE – ACTUALLY, 'CHUCKLES', I JUST, I DON'T DO ANYTHING. I JUST MAYBE, SIT OR SLEEP. THAT'S WHAT. JUST RELAX MYSELF.

INTERVIEWER – WHY DO YOU DO, WHY DO YOU TAKE THAT ACTION?

FEMALE INTERVIEWEE – AT THAT MOMENT MAYBE, MMM, I MIGHT BE WEAK OR, SO BECAUSE OF THAT I HAVE TO RELAX. JUST KEEP MYSELF CALM.

INTERVIEWER – WITH WHO INFORMS YOUR DECISIONS. DO YOU DECIDE YOURSELF OR SOMEONE ELSE?

FEMALE INTERVIEWEE – INTERRUPTS INTERVIEWER, I DECIDED IT MYSELF.

INTERVIEWER – PLEASE BEFORE YOUR DIAGNOSIS, HAVE YOU HEARD, WHAT CONDITION WERE YOU DIAGNOSED WITH?

FEMALE INTERVIEWEE – LUPUS

INTERVIEWER – SO BEFORE THE LUPUS, DID YOU HEAR ABOUT THE CONDITION IN WHICH

FEMALE INTERVIEWEE - 'INTERRUPTS 'MMN MMN. I DON'T HAVE ANY IDEA ABOUT IT

INTERVIEWER – OH OKAY. SO PLEASE WHAT, BEFORE THE DIAGNOSIS WHAT WERE YOU THINKING CAUSED, CAUSE OF THE SYMPTOMS

FEMALE INTERVIEWEE – ACTUALLY I, I WAS THINKING MAYBE, I'VE EATEN SOMETHING WHICH IM NOT SUPPOSED TO EAT OR, OR THE DRUGS BECAUSE I WAS TAKING FERTILITY DRUGS. SO I WAS JUST THINKING ABOUT IT. I THINK THE DRUGS WERE TOO MUCH FOR MY SYSTEM THAT'S WHY I WAS GOING THROUGH THIS THINGS. YH

INTERVIEWER – WHAT ABOUT SPIRITUALLY, YOU THINK SOMEONE HAS SOMETHING IN

FEMALE INTERVIEWEE – 'INTERRUPTS' OH OH, 'CLEARS THROAT', I DO THINK ABOUT THAT BUT I DON'T LET IT BE MY MAIN ISSUE.

INTERVIEWER – OK

FEMALE INTERVIEWEE – YH

INTEVIEWER – PLEASE WHERE DID YOU GO FIRST WHEN THE SYMPTOMS STARTED

FEMALE INTERVIEWEE – [redacted] CLINIC. [redacted]

INTERVIEWER – OH OKAY. HOW WAS THE EXPERIENCE?

FEMALE INTERVIEWEE – ACTUALLY I WAS PREGNANT. WHENEVER I GET PREGNANT I GET A MISCARRIAGE.

INTERVIEWER – HMMM

FEMALE INTERVIEWEE – SO IT WAS THEN I WAS INTRODUCED TO A PHYSICIAN, DR SETH. SO HE MAKES ME, WE DID SOME LABS AND HE GOT TO KNOW THAT I HAVE ERM, S.L.E

INTERVIEWER – SO THAT'S WHEN YOU WERE DIAGNOSED

FEMALE INTERVIEWEE – YES YES. BEFORE I WAS TRANSFERRED TO THIS PLACE

INTERVIEWER – OK

FEMALE INTERVIEWEE – YEAH THAT WAS IN, I THINK IN LAST YEAR NOVEMBER-DECEMBER. NOVEMBER - DECEMBER. SO, ITS BEEN A, ALMOST A YEAR NOW.

INTERVIEWER – OK. SO EERM, WHEN THE SYMPTOMS STARTED, HOW LONG DID IT TAKE BEFORE YOU

FEMALE INTERVIEWEE – 'INTERRUPTS INTERVIEWER' I GOT HERE

INTERVIEWER – YOU GOT TO THE FACILITY? BEFORE YOU WERE DIAGNOSED

FEMALE INTERVIEWEE – YES, WHEN WE DID THE TEST, WE WERENT GETTING THE RESULTS, SO WE WAITED FOR A MONTH BEFORE I WAS TOLD TO COME HERE FOR MEDICATION AND STUFFS. YES, WE WAITED FOR A MONTH.

INTERVIEWER – PLEASE WHAT MADE YOU TO GO TO THAT PLACE.

FEMALE INTERVIEWEE – THAT'S MY CLINIC WHENEVER I GET PREGNANT THAT'S WHERE I GO.

INTERVIEWER – DID YOU VISIT ANY OTHER FACILITY APART FROM THAT PLACE

FEMALE INTERVIEWEE – NO NO. APART FROM POT, I DON'T GO ANY WHERE

INTERVIEWER – WHY

FEMALE INTERVIEWEE – THAT'S WHERE I GO, LIKE, THAT'S MY CLINIC. SO I DON'T VISIT ANY OTHER PLACES

INTERVIEWER – SO NOW THAT YOU'VE BEEN DIAGNOSED, WHAT DO YOU NOW UNDERSTAND ABOUT THE CONDITION?

FEMALE INTERVIEWEE – 'CHUCKLES' ALL I KNOW IS; ALL WILL BE OKAY. THAT'S WHAT THEY TOLD ME, THEY'VE BEEN TELLING ME AND BY GRACE, IVE SEEN IMPROVEMENTS

INTERVIEWER – SO WHEN WE SAY LUPUS, IF YOU ARE TRYING TO EXPLAIN TO SOMEONE, WHAT CAN YOU SAY

FEMALE INTERVIEWEE – I THINK, I THINK ITS ABOUT TISSUE, ITS ABOUT OUR SYSTEMS, THE TISSUES, IT FIGHTS, EERH, OUR TISSUE FIGHTS AGAINST OUR BODY.I THINK IT DAMAGES THE BODY. YH???

INTERVIEWER – SO WHERE DID YOU RECEIVE MOST OF THESE INFORMATION

FEMALE INTERVIEWEE – ACTUALLY HERE. AND IVE BEEN GOING THROUGH THE NET. WHEN I WAS DIAGNOSED, I WENT TO THE NET TO CHECK ONE OR TWO THINGS.

INTERVIEWER – OK

FEMALE INTERVIEWEE – HMMM

INTERVIEWER – SO DO YOU HAVE ANY BELIEVES OF WHAT IS CAUSING THE CONDITION. DO YOU STILL BELIEVE IT'S THE TISSUE OR YOU STILL THINK ITS SPIRITUAL

FEMALE INTERVIEWEE – "INTERRUPTS'' ME I DON'T WANT TO THINK ABOUT THOSE THINGS OO. I GIVE EVERYTHING TO GOD. LAUGHS SUBTLY.

INTERVIEWER – WHY DO YOU LIKE TO TAKE YOUR MIND OFF

FEMALE INTERVIEWEE – I DON'T WANT TO STRESS MYSELF ANYWAY. I DON'T WANT TO STRESS MYSELF SO WHAT IM SUPPOSED TO DO TO TAKE MY DRUGS MAYBE MY PRAYERS MY, THAT'S WHAT I, I DON'T THINK ABOUT ALL THOSE THINGS. IT WILL WORRY ME

INTERVIEWER – SO AFTER THE DIAGNOSIS, CAN YOU TELL US ABOUT YOUR EXPERIENCE AT POT CLINIC.

FEMALE INTERVIEWEE – MMMN. AT [redacted], I WILL SAY THEY WERE JUST TOSSING ME BECAUSE THEY DIDN'T KNOW WHAT WAS GOING ON UNTIL I SAW THE PHYSICIAN. YH. THEY WERE JUST GIVING ME DRUGS PLUS ANTIBIOTICS AND ALL STUFFS BUT, UNTIL I SAW THE OTHER DOCTOR, EVERYTHING WAS OKAY.

INTERVIEWER – SO DO YOU FEEL THE NEED TO VISIT OTHER FACILITIES?

FEMALE INTERVIEWEE – NO NO NO

INTERVIEWER – WHY?

FEMALE INTERVIEWEE – IM OKAY

INTERVIEWER – YOU ARE OKAY

"BOTH *LAUGH BRIEFLY"*

INTERVIEWER – SO WHAT ABOUT TRADITIONAL, HERBAL, MEDICINE

FFEMALE INTERVIEWEE – NO NOT. I WAS TOLD ITS NOT HELPFUL. SO IVE NOT EVEN TEMPTED. AT FIRST I WAS THINKING ABOUT IT BECAUSE OF , YOU KNOW THIS, THEY SAID LIKE TAKING TOO MUCH OF DRUGS, IT AFFECTS OUR IMMUNE SYSTEM AND BUT, A DOCTOR TOLD ME, EERRH, MY HUSBANDS FRIEND, SHE IS A DOCTOR, SHE TOLD ME THE TRUTH THAT I SHOULDN'T TRY, SO, I BELIEVE IN PRAYERS EVERYTHING WILL BE OKAY.

INTERVIEWER – OKAY. SO HOW DO YOU FEEL ABOUT THE OUTCOME.

FEMALE INTERVIEWEE – RIGHT NOW? OH, BY GRACE. EVERYTHING WILL BE EIH, EVERYTHING IS FINE OO.

INTERVIEWER – WHEN YOU SAY FINE, WHAT DO YOU MEAN BY THAT.

FEMALE INTERVIEWEE – OH MY FACE, MY LIKE, EVEN MY MUSCLES, 'CHUCKLES' I WAS REALLY WEAK. I COULDN'T EVEN WALK. BUT NOW I CAN DO EVERYTHING. I CAN MOVE, I CAN DO EVERYTHING BY GRACE.

"BOTH LAUGH BRIEFLY"

INTERVIEWER – SO DO YOU ALWAYS TAKE YOUR MEDICATION AS

FEMALE INTERVIEWEE – OH YES

INTERVIEWER – WHY DO U TAKE IT

FEMALE INTERVIEWEE – I DON'T MISS IT. I HAVE BEEN TOLD TO TAKE IT. THAT WILL HELP ME, SO

INTERVIEWER – SO APART FROM THE PRESCRIBED MEDICATION, DO YOU USE ANY OTHER SELF-HELP PRACTICE

FEMALE INTERVIEWEE – MMN MNN "MEANS NO"

INTERVIEWER – WHAT ABOUT PRAYERS, IT ALSO A SELF-HELP PRACTICE

FEMALE INTERVIEWEE – NOO. I DON'T DO. EIH.

INTERVIEWER – PRAYERS?

FEMALE INTERVIEWEE – AAAH? U SAID PRAYERS? OH EIH, NUMBER ONE. THAT'S MY FOOD I EAT. "*LAUGHS GENTLY"* AS FOR THE PRAYERS DIER? THAT'S WHAT I EAT BECAUSE NOW, BECAUSE OF THESE THINGS IVE LOST MY JOB. BECAUSE OF THE MISCARRIAGES AND OTHER STUFFS, SO, IM JUST DOING SOMETHING ON MY OWN. SO, BECAUSE I DO HAVE TIME TO GO TO CHURCH AND TO PRAY. YH NUMBER ONE.

INTERVIEWER – SO PLEASE WHO KNOWS ABOUT YOUR CONDITION?

FEMALE INTERVIEWEE – MY HUSBAND

INTERVIEWER – ONLY YOUR HUSBAND?

FEMALE INTERVIEWEE – EEEH. AND MY PASTOR AND MY DAD.

INTERVIEWER – YOUR SIBLINGS DON'T KNOW

FEMALE INTERVIEWEE – NOBODY KNOWS AGAIN

INTERVIEWER – SO THOSE WHO KNOW, HOW DO THEY RELATE WITH YOU? HOW HAS THE RELATIONSHIP BEEN

FEMALE INTERVIEWEE – OH. ACTUALLY, WE BELIEVE, IT IS WELL. SO, BECAUSE OF THAT, I DON'T EVEN, THEY DON'T EVEN SEE ME AS A, SOMEONE WHO IS SICK.YH. I WE TRUST GOD THAT EVERYTHING IS OKAY, SO. AND BY GRACE TOO WEVE SEEN MUCH IMPROVEMENT. SO EVERYTHING IS OKAY. IM LUCKY

INTERVIEWER – SO HOW HAS THE CONDITION AFFECTED YOUR ABILITY TO DO THINGS PHYSICALLY. HAS IT AFFECTED YOUR WEAK

FEMALE INTERVIEWEE – OH FOR THAT ONE DIER, ONE THING IVE SEEN ABOUT THIS THING IS YOU DON'T HAVE TO STRESS YOURSELF. EEEIHH. WHEN THE STRESS COMES, HMMM, THEN I HAVE TO SLEEP. I HAVE TO RELAX MYSELF. SO, ONE THING IVE SEEN THAT NO, NO, LIKE YOU DON'T HAVE TO STRESS YOURSELF. THAT'S ALL

INTERVIEWER – BUT EMOTIONALLY. DO YOU FEEL SAD?

FEMALE INTERVIEWEE – EMOTIONALLY, IVE, HMMM, IVE WORKED ON MYSELF BI ERH, “*CHUCKLES”* SO I DON’T LET IT AFFECT ME LIKE EMOTIONALLY OR ….IM OKAY. IM A NORMAL HUMAN BEING. THAT’S HOW I SEE MYSELF AND I DO WHATEVER I WANT TO DO. EVERYTHING IS NORMAL LIKE HOW I WANT

INTERVIEWER – WHAT ABOUT SOCIALLY.? HAS IT AFFECTED YOUR SOCIAL LIFE

FEMALE INTERVIEWEE – OOH NO EEIH. NO OOO. IT DOESN’T. NOW KRAAA MPO I HAVE… “*both laughs briefly”* OH LIKE AT FIRSTWHEN THE SICKNESS CAME, I COULDN’T GO OUT BECAUSE OF MY APPEARANCE.BUT NOW DIER, I CAN DO, I CAN GO WHEREVER I WANT

INTERVIEWER – SO HOW DID YOU COPE WITH ALL OF THIS? TALKING OF SOMETHING TO HOLD ON.

FEMALE INTERVIEWEE – ACTUALLY, I WOULD SAY ITS MY, BY THE GRACE OF GOD AND PRAYERS. BECAUSE AS FOR THE PRAYERS DIER I DIDN’T LEAVE IT OOO. EVERY, EVERY MINUTE, EVERY SECOND.BECAUSE IT WAS SERIOUS. IT WAS SERIOUS. BUT BY GRACE.

INTERVIEWER – DO YOU HAVE HOPE THAT THINGS WILL GET BETTER

FEMALE INTERVIEWEE – EEIIHH. IT IS GOING IN THE NAME OF JESUS. “*laughs”* LUPUS WILL GET OUT OF MY SYSTEM. EIH. FOR SURE. AMEN

INTERVIEWER – AMEN

“*both laugh “*

INTERVIEWER – HOW DO YOU PERCIEVE YOUR FUTURE?

FEMALE INTERVIEWEE – OOOHH. BY GRACE EVERYTHING IS IN THE HAND OF GOD. I KNOW EVERYTHING WILL BE OKAY.

INTERVIEWER –OKAY. THANK YOU VERY MUCH
