# Supplementary material for: Health-seeking behaviour, referral patterns and associated factors among patients with autoimmune rheumatic diseases in Ghana: A cross-sectional mixed method study
Source: PLoS One. 2022 Sep 12;17(9):e0271892. doi: 10.1371/journal.pone.0271892 (PMC9467363; doi:10.1371/journal.pone.0271892)
Supplement: S5 Appendix — (ZIP) [file pone.0271892.s009.zip › AUDIO 31.pdf]

AUDIO 31

Interviewer: Good morning my name is [redacted]

Participant: You're what

Interviewer: [redacted]

Participant: ohhh that's nice my name is [redacted]

Interviewer: ohhh okay so we just want to understand eerrrmm! Your experience with the condition, your knowledge about it and how you're coping

Participant: ohhh okay

Interviewer: so please the first question is what do you do when you're usually not feeling well?

Participant: when I'm not well eerrmmm!

Interviewer: yes

Participant: I change my diet because I think is what I take in that makes me get pains. You know before I was eating meat anything pastries. So when I feel sick. I change it either I become vegan. I hope you know…….

Interviewer: maybe for example

Participant: ehhmm if I was eating a lot of carbohydrate ehhmmm chocolate eehhmmm martins and all that then I go with either I take only fruits, vegetables or even if I will take anything I will just take shrimps

Interviewer: so why do you take that actually?

Participant: eehhmmm I like reading a lot and to lead a healthy life it can reduce the pain before I had swollen feet but when I began vegan like three months the swollen part all when away and I was fine I wasn't limping I wasn't doing anything

Interviewer: so do you do you take that decision on your own or someone else influences you?

Participant: ohh I take it on my own

Interviewer: ohhh! okay ehhmmm before the diagnoses. Please what's your diagnoses?

Participant: cero negative arthritis

Interviewer: ohh okay so did you hear about the condition before?

Participant: No never

Interviewer: so where did you go when the symptoms first started?

Participant: okay so when I was almost out of high school I got a stiff elbow. I couldn't move it.

Interviewer: okay

Participant: so my friend had to force me. So I was getting some funny symptoms, my feet was swollen I was always sick, I was like no. so as usual I went to goggle and typed my symptoms so they gave me options it were like locoes or arthritis. I was scared I thought I had locos. I goggle the best [redacted].

Interviewer: okay

Participant: so I called her personally. I think I called her secretary and I was like I want to come and she said I had to get what is it called transfer

Interviewer: yeah transfer

Participant: I was like I'm [redacted] I have to come and I really want to know what is going on on my body. So I came her booked an appointment. I didn't have any referrals though. Then they run a lot of series of test including HIV, so many things and they noticed that I have cero negative arthritis. So that's what I have started.

Interviewer: that means you came here first

Participant: yeah I came! I came! I didn't even tell my parents I just came.

Interviewer: ohh okay so how long did it take you before you got here?

Participant: eehhmmm I think two weeks

Interviewer: did you visit any facility apart from this place?

Participant: no [redacted] has a pharmacist here so if I tell him he say just take diclo and the pain goes down. I went to some hospital they couldn't see anything and it was strange so I wanted to find out because it was I didn't like how I was feeling

Interviewer: after the diagnoses what do you now know about the condition?

Participant: laughs! I feel is bad eating habit because when I was young I was a little pampered so I don't like fish when I smell it I really want to vomit and all that so even if I take kenkye I want to have it with chicken or some fried beef or something so I think is bad eating habit.

Interviewer: that's what you think

Participant: yeah that's what I think

Interviewer: and where did you receive that information or did you read it somewhere else?

Participant: I read it somewhere else because okay fine I have it my immune system is not good I have it but if I try to take care of myself I think I'm going to live longer or even I won't feel any symptoms

Interviewer: okay

Participant: do you get me

Interviewer: so what did you read about the diet?

Participant: so I read about what you should eat if you have arthritis and like the good living you have to adopt and I like exercising a lot ehhmmm. I don’t take any soda stuff like coke, what do they all it sprite and those things. I rather take ginger juice, or orange like the natural on I don’t even like the paper one.

Interviewer: so most of the information you got it online?

Participant: yeah I got it online

Interviewer: so do you have any believe system towards the condition do you think is spiritual?

Participant: my mom was saying is spiritual so for some time I stopped taking my medication and I was like my feet is gone. So I started feeling the pain again and I was like the pain is back. You know what I was going to the the ‘’F’’ word. I will just stick to my medicine. Now I take just one drug first I use to take five drugs or six. Now I take just a pile a day now I think is fine. Usually we always be thinking something is spiritual that’s why Ghanaians are not going further. So I don’t even want to think about that.

Interviewer: so after the diagnoses have you thought of the need to go to any herbal

Participant: I did. They call it organic you can imagine the name right

Interviewer: yeah

Participant: so I went there and they said they want to flush my body so they gave me some very bitter stuff and they said I shouldn’t eat carbohydrates so I was taking…. I was actually suffering that’s why I even became vegan. I had no carbohydrates in my system so even if I did maybe banana or something could give me carbohydrate. I noticed the pain was even more. They were giving me drugs though but everything was organic. The pain was more every time I’m sick I will be crying, when I go to work I have to go back home. So I stopped and I was spending almost 3000 for two weeks. No for a day I can spend like 2000. The next two week their like I have to come I was like I can’t do this I have to stop because me not taking some of the vitamins was making me sick more. So I decided to stick to my clinic now at Korle-bu.

Interviewer: how do you compare clinic here in Korle-bu to other places?

Participant: I think is way better because if I don’t tell you I’m sick you won’t know I’m sick.so I think is better

Interviewer: and how do you feel about it and the current outcome now?

Participant: from here?

Interviewer: yeah

Participant: is good is good. Even if I’m not feel well I know is my fault because I’ve been eating stuff I’m not supposed to eat so I think so far the drugs are good. I don’t see any side effect.

Interviewer: so do you take your medication

Participant: yeah I have to

Interviewer: why?

Participant: I need to. Laughing!!! I think is a need I need it

Interviewer: so apart from the prescribed medication do you use any self-help practice?

Participant: noo apart from my eating habit and my exercises

Interviewer: ehhh sometimes you include prayers

Participant: yeah I always pray ohh god I don't want to get weak .i don't even get weak. If I do I know is something I'm not doing right. So I need to change it.

Interviewer: who knows about your condition?

Participant: eehhmm no everybody but almost everybody if you're my friend and you're very close to me. You know people are very curious?

Interviewer: yes

Participant: I came to hospital every three month. You'll think maybe I have some serious illness. So I just tell them I have negative arthritis some people even google and search for it. Imp not shy at all.

Interviewer: so how is the relationship between those who know about it?

Participant: for dating some people get tired because sometimes you complain my headaches and all they get tired of it. I think is cool I don't care.

Interviewer: how has it affected your social life?

Participant: I actually don't care because even if I have to date I have to make friends. You have to accept me the way I am. Is not like I'm going to die today or something. I don't care. I really don't care.

Interviewer: so physically how are you? So physically how has it affected you?

Participant: I don't know sometimes I get rushes on my skin or my eyes

Interviewer: okay so what about emotionally how has it affected you? Is it that bad?

Participant: it used to. When I was diagnosed I use to cry a lot because sometimes I get headaches and is that bad and I start crying but I think I'm fine.

Interviewer: so with all this experience how do you deal with them?

Participant: I just go with the flow. Yeah I have it. I just go with the flow. If I'm gone today fine.

Interviewer: is that that something that motivates you

Participant: yeah because ehhmmm when I started coming here some few months I wanted to do a pageant but I was really in pain. I said to myself I really want to do this. Sometimes we had to be on our feet for hours I felt pain but it made me relax. Sometimes I got tired I ask myself why I'm I even in this pageant but I still pushed on and I went to finals and I think I was proud of myself and I think my doctor also.

Interviewer: thank you very much …
