# Supplementary material for: Health-seeking behaviour, referral patterns and associated factors among patients with autoimmune rheumatic diseases in Ghana: A cross-sectional mixed method study
Source: PLoS One. 2022 Sep 12;17(9):e0271892. doi: 10.1371/journal.pone.0271892 (PMC9467363; doi:10.1371/journal.pone.0271892)
Supplement: S5 Appendix — (ZIP) [file pone.0271892.s009.zip › AUDIO 32.pdf]

AUDIO 32

Interviewer: what do you do when you are not well?

Participant: the medications they give me is what I always take. Then when it is time for my review I come

Interviewer: why do you take that measure?

Participant: the illness started with joint pains and I was brought here and given medications. So that why I take the medications

Interviewer: is that why you take the medications?

Participant: yes but they have taken some out. I'm now left with my BP medications

Interviewer: do you take the decision on your own or someone does?

Participant: no, I come to the hospital then they ask me to go to the chemical store for the medication.

Interviewer: What do you know about the sickness you have been diagnosed of?

Participant: Erm, I have no idea, but these drugs speak more of the illness.

Interviewer: Before you were diagnosed, did you know anything about the illness?

Participant: No, I was even surprised that people could actually have the same illness like mine or if it existed.

Interviewer: When you started experiencing the symptoms, where was your first point of contact?

Participant: I went to a clinic at my place [redacted] then I travelled to [redacted]. Then went to clinic which referred me to Korle-bu. The clinic is [redacted]

Interviewer: How long did it take to receive medical assistance?

Participant: It took just two day or a day and whenever it happens, the pains were felt in my joints and the next thing done is to visit the nearest hospital.

Interviewer: When you started experiencing the pains in your joints, did you visit a prayer camp?

Participant: No, I did not. As for prayer camp I do go there but not because of the illness. I decided to visit the hospital first before doing any other thing.

Interviewer: Has anyone explained further in terms of what your illness is all about?

Participant: Not really, they only put me on medication but no one has told my anything. But I have asked the doctor.

Interviewer: so you don't know the cause?

Participant: yes

Interviewer: So, what do you think is the cause of this illness?

Participant: well, I can't tell. The illness just came so I can't tell.

Interviewer: So, what do you think that someone has caused it to happen?

Participant: Not really, I don't believe in those things it's just and illness and we just have to pray about it. That is what I believe. I don't believe anyone caused it.

Interviewer: What about ageing being the cause of the illness?

Participant: Yes, I sometimes think of it as the reason and also because the age I have gotten to may have caused the illness. I am 61 and as am growing older, these symptoms would submerge.

Interviewer: Did it ever occur to you, to administer herbal medicine to cure the illness.

Participant: No please. I don't leave close by so I wanted to, now that I feel normal and the symptoms have reduce, I want to go back to the old clinic

Interviewer: What has been your observation concerning help administered in both health facilities? Are there any differences?

Participant: yes a lot. With the labs I have to go to other places to do it but it is not so here

Interviewer: and how do the doctors treat you?

Participant: they treat me well. The ask me questions then I answer

Interviewer: so now how do you feel?

Participant: I feel very fine. I can work with it. I am a driver and I can still work. When they give me three months I just come and take my medication. As for my medications I don't joke with it.

Interviewer: I was about to ask if you take the medications they give you

Participant: the illness is painful and I don't want it to come back. And it worries me so I have to take it.

Interviewer: apart from the medications the doctor give you do you take other things?

Participant: no, they check the medications for me every three months then I come for it every month.

Interviewer: so do you include the exercise?

Participant: no

Interviewer: What about prayer?

Participant: yes I pray. Because God first so you to pray in addition to taking your medications

Interviewer: who have you told about your illness?

Participant: my wife and family

Interviewer: did anything change afterwards?

Participant: no, everything is ok

Interviewer: so do they help you?

Participant: yes they do

Interviewer: so has the illness affected you? Like physical, how you do things?

Participant: when it comes I can’t do anything but when it hasn’t come…. i can do anything I want

Interviewer: do you think about it?

Participant: I use to think about it before. But now I don’t.

Interviewer: do you feel sad?

Participant: no

Interviewer: do you hang out with your friends? Do you go out?

Participant: yes I do. They are sometimes surprised I am still active

Interviewer: so what encourages you?

Participant: God. I believe in God so I know that everything is ok. I use to think about it a lot because I have not seen anything like this before. Someone just becoming ill. But this time it is okay
