# Supplementary material for: Health-seeking behaviour, referral patterns and associated factors among patients with autoimmune rheumatic diseases in Ghana: A cross-sectional mixed method study
Source: PLoS One. 2022 Sep 12;17(9):e0271892. doi: 10.1371/journal.pone.0271892 (PMC9467363; doi:10.1371/journal.pone.0271892)
Supplement: S5 Appendix — (ZIP) [file pone.0271892.s009.zip › AUDIO 36.pdf]

Audio 36

Interviewer: please what do you do when you are usually not feeling well?

Participant: eeerrrm. I prefer to browse the internet.

Interviewer: You prefer to browse the internet

Participant: Yes. Yes

Interviewer: Why do you take that decision?

Participant: Not any particular reason, but at least to help me to, eeerrrmmm, feel a little happiness because browsing is very nice to me. Which I really really browse a lot when I'm in a sad mood

Interviewer: So do you take that decision on your own or someone influences your decisions?

Participant: Not at all. Personal decision

Interviewer: Please what's your current diagnosis?

Participant: Eeerrrmmm. Mine has been changing changing. First they told me its somatic arthritis. Now they told me it's something something active arthritis which is equal to or similar to R.A, rheumatoid arthritis. So in fact, i personal don't know. What i knows it's an arthritis.

Interviewer: So before the diagnosis did you hear about the condition?

Participant: No no. Yes i knew arthritis but not in detailed

Interviewer: Please what did you know about it?

Participant: Eerrrmm normally what i thought it was pertaining to old people. Old age until i personally experienced and know that it can be associated with any person. Even child and grown-ups as well.

Interviewer: So where did you get that information from?

Participant: Eerrrm. From the net.

Interviewer: Ok. So. eeerrrrrm. When the symptoms started where did you go first?

Participant: So the first, eerrrmm. [redacted] polyclinic was the first at the initial stage then, errm, for almost a year, they told me probably it could be sickle cell so i should go and do a sickle cell test and nothing happened so during that, that period we went to [redacted] polyclinic as well and we were referred here from [redacted] polyclinic

Interviewer: So how long did it take from the first symptom to the first facility? That's mamprobi polyclinic

Participant: Like one year. Yeah. One year.

Interviewer: So can you tell me a bit about, what prompted you sorry, to go to the facility

Participant: Okay so we thought it was normal sickness so you go and they give you these malaria and those things so maybe probably two weeks three weeks, you will be feeling better, but it was getting worser and worser so my parents took me to errm, [redacted], then i was transferred to come and see a specialist here

Interviewer: So did you visit any other facilities. Maybe herbal, or prayer camps?

Participant: Not at all

Interviewer: Why didn't you?

Participant: I personally do not prefer those things

Interviewer: So now that you've been diagnosed, you know at least a bit about the condition?

Participant: Yes

Interviewer: What do you understand by the condition?

Participant: Ok. So i know that it is an auto immune deficiency which also erm, is a chronic disease so we have to manage our lifestyles and those things

Interviewer: Ah. Okay. And where did you receive that information ?

Participant: Ok so erm, we have these support group, "rheumatoid, chuckles, the full name is,

Interviewer: Tri?

Participant: Exactly. Something like that. So I've been there for, but not really, an active member. But at least, they help us understand it well and a whatsapp group that give us a little information about it.

Interviewer: Personally what believes do you have about it. Do you think its spiritual aspects or aging could also cause or you think lifestyle. Has your mind ever gone to that direction?

Participant: As in

Interviewer: The actual cause of this. Do you think spiritual or the symptoms? Do you think?

Participant: Oh okay.so me my believe is very simple. What you cant see, don't believe it, that's all.so far as i don't see those spirits, i don't believe in spirits but i just believe in God although God is spirit. So that's the only spirit being i believe in. The rest i can't see so i don't believe in it.

Interviewer: So can you tell us about the experience of the other facilities before you got here. How was the experiences?

Participant: Okay. It was normal. Normal lab tests. Normal xrayand those things.it was normal hospital checkups.

Interviewer: After, eerrh, for now that you are here, do you feel the need to visit any other treatment facility?

Participant: Not at all

Interviewer: Why?

Participant: Okay. Yes yes. If Jehovah God permits me, and i have the money, i would prefer to go out for advanced treatment. But not like to change to like herbal those things. Yes. I prefer these kinds of medicines

Interviewer: How do you feel about the outcome?

Participant: Pretty good. At first it was worse, like i told you, but now, unless i tell you i have a chronic disease, you will never know.

Interviewer: Do you always take your medications as prescribed?

Participant: Always. Yes. Cause if you don't, "laughs shabbily". In fact, it like, it's like life support, so immediately. Okay not always, not not for three days, that one dier you will feel the effects immediately but as least a day you can forget it. But regularly, yes.

Interviewer: So apart from the prescribed medication, do you use any self-help practice?

Participant: Not at all

Interviewer: Do you rely on prayers too?

Participant: Yes yes. Of course. Yes. I do rely on prayers too but besides that, the rest forget it.

Interviewer: Why do you rely on prayers?

Participant: Because i believe in God. And with God, everything is possible.

Interviewer: Who have you told about your condition?

Participant: My doctors

Interviewer: Your doctors? Your family?

Participant: Ooohh. Noo. If you don't know, you don't know. That's how it is.

Interviewer: And how has the relation being? Those who know about it

Participant: I don't even know. Because i am not that type of person who prefers to because if i tell you, there's nothing that you can do to it. So if you know, you know. If you don't know, that's all

Interviewer: So how has it affected your ability to operate, physically?

Participant: Mmmn. At first, i mean i can’t walk, at least, a little then you will feel tired and those things. But now everything has changed. I can do whatever i used to do previously. So now it’s okay. With my activity, it does not have any impact, curren5tly, on my activity.

Interviewer: So do you think about it?

Participant: Mmmnnn. Not at all.

Interviewer: It doesn’t make you feel sad or

Participant: Not even a second

Interviewer: Sad or fear about

Participant: At first but currently, no

Interviewer: Socially, do you hang out the way you used to hang out?

Participant: I personally don’t. “Chuckles”. Anti-social so i don’t think it’s part of the condition but i personally don’t feel like doing those things, because normally it comes along with cost and i hate anything that comes with cost.

Interviewer: So how do you cope with the symptoms?

Participant: Eerrrmm. In fact, it has become used to my normal routine, so ive become used to, yaeh, so i don’t do jogging, football and all that you will feel pain so, i don’t do that. yeah. In fact in coping, i have changed have adopted to my lifestyle so I’m okay with that. Yeah.

Interviewer: Thank you very much. Laughs briefly.
