# Supplementary material for: Health-seeking behaviour, referral patterns and associated factors among patients with autoimmune rheumatic diseases in Ghana: A cross-sectional mixed method study
Source: PLoS One. 2022 Sep 12;17(9):e0271892. doi: 10.1371/journal.pone.0271892 (PMC9467363; doi:10.1371/journal.pone.0271892)
Supplement: S5 Appendix — (ZIP) [file pone.0271892.s009.zip › AUDIO 35.pdf]

AUDIO 35

PARTICIPANT: Me, I can't speak English

INTERVIEWER: We will manage with the Twi, hehehe

INTERVIEWER: What do you do when you are not well?

PARTICIPANT: I take my drug and sleep,

INTERVIEWER: Why do you do that?

PARTICIPANT: The drug is what controls the illness, so when I take the drug and sleep for an hour or two I feel much better.

INTERVIEWER: Did you take the decision by yourself or someone else influenced you to do it?

PARTICIPANT: well, a drug has been prescribed to me and I have been advised that the drug will help to calm the illness, so when I take in the drug and eat well I feel ok?

INTERVIEWER: what condition have you been diagnosed of?

PARTICIPANT: I also feel pains in my joint, so I will say rheumatoid arthritis and lupus erythematosus and I have also been attacked by my lungs.

INTERVIEWER: Before you were diagnosed did you ever hear about this condition?

PARTICIPANT: I was taken to lots of places, some even concluded that it was a spiritual condition.

INTERVIEWER: Where did you first go when you saw the symptons?

PARTICIPANT: I went to church because they were concluding that the symptons of paralysis was a spiritual case. So the church was the first place we went to.

INTERVIEWER: How many weeks and months did it take since it started before going to church?

PARTICIPANT: It started in April like Typhoid and malaria, but I later realized that it wasn't that, so was taking in typhoid and malaria drug and I realized that I wasn't dealing with the right illness, so I spent the month of May, June, July, August at the church and I was later referred to [redacted]

INTERVIEWER: Why did you go to church?

PARTICIPANT: I was told it was a spiritual illness, and you could realized that out of ignorance, because they realized the symptons they concluded that I was sent to the spiritualist.

INTERVIEWER: what do you mean by they?

PARTICIPANT: like my mum, family and those around me, they didn't know the exact cause of the illness because the diagnosis for malaria and typhoid always proved negative. So they concluded that it was a spiritual illness.

INTERVIEWER: Now that you have been diagnosed and you know what exactly is wrong with you, how do you understand it?

PARTICIPANT: I know this an illness and as the doctors have claimed to manage me, I have decided to permit it.

INTERVIEWER: Personally what do you think is the cause of your illness?

PARTICIPANT: I see it as excess infection, apart from that I don't see anything causing this.

INTERVIEWER: Do you agree with your family that this is a spiritual issue?

PARTICIPANT: No I don't agree with them.

INTERVIEWER: Can you tell about your experiences from where you went, both from the hospital and the church.

PARTICIPANT: For the church the only thing they give is water and oil to drink but the more the prayer you still find yourself facing the same condition. But with the hospital you are given pain killers and drugs, so you feel much better.

INTERVIEWER: After coming here, would you want to visit another place for treatment?

PARTICIPANT: No

INTERVIEWER: So how do you see the outcome?

PARTICIPANT: Frankly speaking, the first day I came in, I was fortunate to meet [redacted] i have recovered with the drugs she gives you and its as if I never felt sick. I can move and do everything so well. But its been a year now since my lungs disturbed coupled with some infections and that is what is making me weak but I feel healthy when i feel strong when I take the drugs.

INTERVIEWER: So do you take the drugs as prescribed to you and how do you see the drug that has been prescribed to you?

PARTICIPANT: The doctor is the one who has run the test and he is aware of the quantity to be taken to fight the illness. So I take the medicine according to the prescription of the doctor.

INTERVIEWER: Do you take any other drug in addition to the drugs prescribed to you?

PARTICIPANT: No I don't.

INTERVIEWER: Do you add to the medication prayer?

PARTICIPANT: For prayers we always pray, as a Christian you can't do without it. But I personally do not pray concerning the illness because I see it to be an illness I just contacted so if it's the will of God he will touch and heal you.

INTERVIEWER: Who have you spoken to concerning this illness?

PARTICIPANT: I have spoken to my work mates and the people around me including my family and friends.

INTERVIEWER: How do they relate to you now after the illness?

PARTICIPANT: They relate well to me, I never knew they loved me like that, I realised it after this illness. There are days I find it difficult to wake up, my mum lifts me up, she assists me with all that I do, they feed me, they help me with all things.

INTERVIEWER: So how has the condition affected you physically?

PARTICIPANT: it has affected my looks, the way I walk, my speech, it has affected me in all that I do.

INTERVIEWER: So do you think about it?

PARTICIPANT: I really do think about it, I find it difficult going to work, I was sacked from work after the sickness affected my lungs. Because they realized I couldn't work effectively again.

INTERVIEWER: So does it make you worried?

PARTICIPANT: it makes me worried, but i know I will certainly be able to work when I am fine.

INTERVIEWER: So socially, how has it affected you?

PARTICIPANT: I don't go out, I lay in bed all day.

INTERVIEWER: What inspires you?

PARTICIPANT: I inspire myself with the fact that I wasn't looking like this and that I know I will certainly be ok, when I see other patients, they look ok so I know I will also be better one day
