# Supplementary material for: Health-seeking behaviour, referral patterns and associated factors among patients with autoimmune rheumatic diseases in Ghana: A cross-sectional mixed method study
Source: PLoS One. 2022 Sep 12;17(9):e0271892. doi: 10.1371/journal.pone.0271892 (PMC9467363; doi:10.1371/journal.pone.0271892)
Supplement: S5 Appendix — (ZIP) [file pone.0271892.s009.zip › AUDIO 34.pdf]

AUDIO 34

INTERVIEWER: What do you do when you are not feeling well?

PARTICIPANT: I go to the hospital

INTERVIEWER: Why do you go to the hospital?

PARTICIPANT: I feel self - medication is not good, so when you realize you are not well you need to go the doctor so he prescribes a medicine for you, so when you go for the lab test you can be given the right medicine.

INTERVIEWER: Please did you take the decision to come to the hospital yourself or someone influenced that decision?

PARTICIPANT: I personally took that decision.

INTERVIEWER: What is you diagnosis**?**

PARTICIPANT: Rheumatoid Arthritis

INTERVIEWER: Have you ever been informed about this illness before you were diagnosed of it?

PARTICIPANT: I had no knowledge about arthritis until I was diagnosed.

INTERVIEWER: Where did you first go when the symptoms started?

PARTICIPANT: I went to [redacted] and I was told it was malaria. And I later went to [redacted] and I was told typhoid, I was later referred to [redacted] before coming finally to the main Korle bu,that was where I came to realize that it was arthritis.

INTERVIEWER: How long did the symptoms start before you visited the first facility?

PARTICIPANT: When I wake up I usually feel pains in my joints and knees that when I realized that I wasn’t fine. So I took like a month before visiting the facility.

INTERVIEWER: So you visited the facility because of the symptoms?

PARTICIPANT: I went there because of the symptoms, I had a swollen knee, joint pains, my feet was also swollen, and the pain were in all part of the joints.

INTERVIEWER: Did you visit any herbal or traditional facility?

PARTICIPANT: I tried the herbal, but unfortunately I didn't any improvement because when I take in the herbal medicine the pain gets intense so I stopped taking the herbal medicine.

INTERVIEWER: Now that you have been diagnosed do you now understand the condition?

PARTICIPANT: A bit, but they still don't know what causes this condition, but when they make mention of it I know its joint pains.

INTERVIEWER: Where did you get this information from?

PARTICIPANT: I was informed about this when I came to the clinic.

INTERVIEWER: So personally what do you think causes this condition, is it as a result of ageing or it's a spiritual condition?

PARTICIPANT: Well, I don't know.

INTERVIEWER: Or you feel you are going through this because of what you have done?

PARTICIPANT: I am not sure

INTERVIEWER: After you have been diagnosed would you want to go elsewhere?

PARTICIPANT: No, because for korle bu they have lots of machines so when you are diagnosed of an illness and given a medication you only have to take the medication.

INTERVIEWER: What difference have you realized between korle bu and the other facilities?

PARTICIPANT: Korle bu is more advanced so before they inform you about the ailment, they would have taken you through series of tests.

INTERVIEWER: So how do you feel about the outcome?

PARTICIPANT: It's ok. But certain times the pain gets a bit intense.

INTERVIEWER: Do you take the medicine as prescribed?

PARTICIPANT: Yes I do, but I was influenced by someone to take another medication some time ago, but after taking in that medicine I realized it wasn't helping my condition. The medications given is very effective when taken it manages the pain. It's ok

INTERVIEWER: Apart from prescribed medication do you use any other thing?

PARTICIPANT: I don't use any other thing?

INTERVIEWER: What about prayer?

PARTICIPANT: I pray because I know God can do all things, so after taking the medicine I ask him to show me his mercy, because each drug has its own side effect. So God is the only we are appealing to show us his mercy and to heal us of this illness.

INTERVIEWER: Apart from the medication, do you exercise?

PARTICIPANT: I don't exercise but I walk. For me I am affected at all my joints so it makes me tired. If I can't exercise I walk.

INTERVIEWER: Who knows about your condition?

PARTICIPANT: My family knows about my condition.

INTERVIEWER: How do they relate to you?

PARTICIPANT: They relate very well to me, in fact they have been of support to me, especially my parents, they assist me in getting my medication and I have been getting financial assistance from people as well to buy the drugs.

INTERVIEWER: How has the condition affected you physically?

With how you walk, house chores?

PARTICIPANT: Apart from walking I cannot do any other thing.

INTERVIEWER: Do you think about it?

PARTICIPANT: I really think about it a lot because it's difficult that as a young girl you will suffer this condition, which will restrict your movement. And even as you can't move about you still feel pain as you are seated. It makes you think a lot.

INTERVIEWER: Does it make you scared or sad?

PARTICIPANT: I really feel sad

INTERVIEWER: In terms of relationship, how has it affected you?

PARTICIPANT: When you are ill for a long period everyone neglects you, so I don’t have anyone now.

INTERVIEWER: So what inspires you in spite of all these?

PARTICIPANT: I usually listen to preaching, and this helps me a lot. There are certain times the pain becomes intensive and you wouldn’t know what to do so as you pray and study the word of God it also helps me.
